# Cell Fate Decisions in Malignant Hematopoiesis: Leukemia Phenotype Is Determined by Distinct Functional Domains of the MN1 Oncogene

**DOI:** 10.1371/journal.pone.0112671

**Published:** 2014-11-17

**Authors:** Courteney K. Lai, Yeonsook Moon, Florian Kuchenbauer, Daniel T. Starzcynowski, Bob Argiropoulos, Eric Yung, Philip Beer, Adrian Schwarzer, Amit Sharma, Gyeongsin Park, Malina Leung, Grace Lin, Sarah Vollett, Stephen Fung, Connie J. Eaves, Aly Karsan, Andrew P. Weng, R. Keith Humphries, Michael Heuser

**Affiliations:** 1 Terry Fox Laboratory, BC Cancer Agency Research Centre, Vancouver, BC, Canada; 2 Department of Medicine, Faculty of Medicine, University of British Columbia, Vancouver, BC, Canada; 3 Department of Laboratory Medicine, Medical School of Inha University, Incheon, Korea; 4 Department of Internal Medicine III, University Hospital of Ulm, Ulm, Germany; 5 Institute of Experimental Cancer Research, Comprehensive Cancer Centre, University Hospital of Ulm, Ulm, Germany; 6 Department of Pediatrics, Cincinnati Children's Hospital Medical Center, Cincinnati, Ohio, United States of America; 7 Department of Medical Genetics, University of Calgary, Calgary, AB, Canada; 8 Institute of Experimental Hematology, Hannover Medical School, Hannover, Germany; 9 Department of Hospital Pathology, Catholic University of Korea, Seoul, Korea; 10 Genome Sciences Centre, BC Cancer Agency, Vancouver, BC, Canada; 11 Department of Pathology and Laboratory Medicine, University of British Columbia, Vancouver, BC, Canada; 12 Department of Hematology, Hemostasis, Oncology, and Stem Cell Transplantation, Hannover Medical School, Hannover, Germany; Queen's University Belfast, United Kingdom

## Abstract

Extensive molecular profiling of leukemias and preleukemic diseases has revealed that distinct clinical entities, like acute myeloid (AML) and T-lymphoblastic leukemia (T-ALL), share similar pathogenetic mutations. It is not well understood how the cell of origin, accompanying mutations, extracellular signals or structural differences in a mutated gene determine the phenotypic identity of leukemias. We dissected the functional aspects of different protein regions of the MN1 oncogene and their effect on the leukemic phenotype, building on the ability of MN1 to induce leukemia without accompanying mutations. We found that the most C-terminal region of MN1 was required to block myeloid differentiation at an early stage, and deletion of an extended C-terminal region resulted in loss of myeloid identity and cell differentiation along the T-cell lineage in vivo. Megakaryocytic/erythroid lineage differentiation was blocked by the N-terminal region. In addition, the N-terminus was required for proliferation and leukemogenesis in vitro and in vivo through upregulation of *HoxA9*, *HoxA10* and *Meis2*. Our results provide evidence that a single oncogene can modulate cellular identity of leukemic cells based on its active gene regions. It is therefore likely that different mutations in the same oncogene may impact cell fate decisions and phenotypic appearance of malignant diseases.

## Introduction

The postulated requirement for induction of leukemogenesis has long been the combination of class I and II mutations [1], although recent insights into the genetic composition of acute myeloid leukemia (AML) cells has revealed additional pathogenetic mechanisms including changes in epigenetic regulation. On average, 13 coding genes are mutated per AML genome [2], suggesting that several events are required for leukemogenesis. Despite the heterogeneity of cells that can give rise to AML, only a small proportion of AML cells show clonogenic activity in culture and only a small fraction of AML blast cells are able to confer disease to immune-deficient mice [3]. While such disease-propagating or leukemia-initiating cells (LICs) may be rare, they are not necessarily restricted to the most primitive cells within the hematopoietic hierarchy but rather, can include committed progenitor cells such as common myeloid progenitors (CMPs) or common lymphoid progenitors (CLPs) [4]–[7]. The high level of heterogeneity seen in AML and within an individual patient underscores the importance of understanding the molecular mechanisms underlying this disease and the functional consequences on leukemic cells.

While there is a high degree of cellular heterogeneity within the cells of an individual leukaemia [8], there is striking redundancy of mutated genes in distinct diseases like AML [9], T-lymphoblastic leukemia (T-ALL) [10], and primary myelofibrosis [11], including mutations in *DNMT3A* and several other genes. Explanations for how mutations in the same gene can cause different diseases may include: differing cells of origin [12] or cell-extrinsic signals [13], as illustrated by the ability of the MLL-AF9 fusion gene to cause myeloid and lymphoid leukemias; the influence of the microenvironment, such as the ability of abnormal stroma cells to induce myelodysplasia in hematopoietic stem cells (HSCs) [14]; and the ability of mutations to change the lineage potential of the oncogene and possibly the phenotype of the disease, as in EZH2 mutations in B-non-Hodgkin lymphoma and myeloid disorders [15], [16]. The meningioma (disrupted in balanced translocation) 1 (*MN1*) model of leukemogenesis constitutes a simple and ideal model to test this latter hypothesis due to its ability to induce leukemia as a single hit through constitutive overexpression [17].

The ability of MN1 to induce rapid onset leukemia on its own highlights its central regulatory role in hematopoietic transformation. MN1 has been shown to be most highly expressed in murine CMPs, but is downregulated upon differentiation [17] and is capable of enhancing proliferation of human CD34+ cord blood cells [18]. High MN1 expression has been associated with both acute myeloid and lymphoid leukemias [19] as well as other AML characteristics such as inv(16) [20] or overexpression of EVI-1 [21]. Significantly, it also has been identified as an independent prognostic factor in patients with AML with normal cytogenetics, associated with shorter relapse-free survival, overall survival, and resistance to ATRA-induced differentiation [19], [22]–[25]. As loss of MN1 expression has been shown to impair proliferation and significantly decrease clonogenic activity of human leukemic cells, it is a potential therapeutic target in AML patients [26].

MN1 has been shown to rapidly induce leukemia in mice [20], [22]. We have recently shown that MN1 is capable of transforming single CMP cells as the cell of origin [17]. Significantly, GMPs required co-overexpression of *Meis1* for in vitro transformation, and the additional co-overexpression of HOXA9 or HOXA10 to induce leukemia in vivo [17]. Loss of MEIS1 expression abrogated leukemic activity in MN1 cells, suggesting that, combined with co-localization of MN1 and MEIS1 at a large proportion of MEIS1 target sites, MEIS1 and its cofactor HOXA9 are essential to MN1 leukemogenesis [17]. In addition, MN1 cells are arrested at an immature stage of myelopoiesis and are highly resistant against all-trans retinoic acid (ATRA) [22], a potent inducer of myeloid differentiation, although ectopic CEBPα expression, which MN1 is thought to repress, can abrogate the leukemogenic activity of MN1 [18].

We hypothesize that multiple functions are encoded in this protein and can be localized to different regions. Thus, delineation and localisation of these functions at a structural level will provide insight into the key mechanisms required for leukemic transformation by a single central regulator such as MN1. Despite the established role of MN1 overexpression in leukemia, little is known about the protein itself. The MN1 protein is highly conserved between different species, but largely lacks recognised protein domains excepting two proline-glutamine stretches and a single 28 residue-long glutamine stretch. Here, we systematically localise known properties of MN1 leukemia using both *in vitro* and extensive *in vivo* studies to specific physical regions of wildtype MN1 through a detailed structure-function analysis of MN1. We demonstrate that the proliferative ability and self-renewal activity, and the inhibition of megakaryocyte/erythroid, myeloid, and lymphoid differentiation are localised to distinct regions within MN1 and provide evidence that different mutations of a single oncogene can induce distinct diseases such as myeloid and lymphoid leukemia and myeloproliferative disease.

## Materials and Methods

### Retroviral vectors and vector production

Retroviral vectors for expression of MN1 [22] and NUP98HOXD13 (ND13) [27] have been previously described. Primers were designed for each MN1 mutant truncation construct to ensure the N- and C-termini of the final construct were flanked by *Not*I sites, then subcloned into the expression vector pSF91 [28] upstream of the internal ribosomal entry site (IRES) and the enhanced green fluorescent protein (GFP) gene. MN1 Strategy 1 constructs were generated by PCR amplification of the N- (proximal) and C-terminal (distal) regions of the construct with *Hind*III sites at the internal sites. The proximal and distal fragments were then subcloned into the pSF91-IRESeGFP vector. As a control, the pSF91 vector carrying only the IRES-enhanced GFP cassette was used. Constructs were validated by sequencing and correct expression and transmission were confirmed by qRT-PCR and PCR. Primer sequences can be found in Table S1. For HA-tagged constructs, full-length and MN1 mutant deletion constructs were cut to ensure the N- and C-termini of the final construct were flanked by *BglII* or *BamHI* (for constructs lacking the N-terminal region) and *NotI* sites, respectively, then subcloned into the MSCV-IRES-GFP expression vector [29], and an HA-tag was cloned to the N-terminus of MN1 or the deletion constructs. Helper-free recombinant retrovirus was generated by using supernatants from the transfected ecotropic Phoenix packaging cell line to transduce the ecotropic GP + E86 packaging cell line [30].

### Clonogenic progenitor assays

Colony-forming cells (CFCs) were assayed in methylcellulose (MethoCult M3434 or MegaCult-C, Catalog No. 04964; STEMCELL Technologies, Vancouver, BC, Canada). For each assay freshly isolated and transduced unsorted bone marrow cells were plated in duplicate in Methocult medium (1000 cells/well). Colonies were evaluated microscopically 10 days after plating using standard criteria. To assay megakaryocyte progenitor frequency, freshly isolated and transduced bone marrow cells were sorted for GFP expression, and 1×10^5^ cells were suspended in MegaCult-C medium containing recombinant human thrombopoietin (50 ng/mL), recombinant human IL6 (20 ng/mL), recombinant human IL11 (50 ng/mL), and recombinant mouse IL3 (10 ng/mL), mixed with collagen and dispensed in chamber slides (all from STEMCELL Technologies, Vancouver, BC, Canada). Cultures were incubated at 37°C for 7 days. Slides were stained with acetylthiocholiniodide according to manufacturer's instructions, and colonies were counted manually under a microscope, as previously described [31].

### Quantitative real-time RT-PCR

Total RNA from stored, frozen cell pellets was isolated using TRIZOL reagent (Life Technologies, Burlington, ON, Canada). Total RNA was converted into cDNA using the SuperScript VILO cDNA synthesis kit (Life Technologies, Burlington, ON, Canada) using 500 ng of total RNA. Quantitative real-time PCR was performed as previously described using the 7900 HT Fast Real-Time PCR system (Applied Biosystems, Foster City, CA, USA) [32] and Fast SYBR Green Master Mix (Life Technologies, Burlington, ON, Canada) [32]. Relative expression was determined with the 2^−ΔΔCT^ method, and the housekeeping gene transcript *Abl1* was used to normalize the results. Primers were manufactured by Life Technologies. Primer sequences can be found in Table S2.

### Western blot analysis

For Western blot analysis, 1×10^6^ cells were lysed with 150 µL lysis buffer (50 mM Tris-HCl [pH 8], 0,1% Tween-20, 0.1% SDS, 150 mM NaCl, 0.5 mM EDTA, 10 mM DTT, and 1 mM PMSF, plus protease inhibitor cocktail; Sigma, Oakville, ON, Canada) and incubated for 20 minutes on ice. NuPage LPS loading buffer (4x) and NuPage Sample Reducing Agent (10x) (Life Technologies, Burlington, ON, Canada) were added and samples were heated for 15 minutes at 95°C. Lysates were loaded onto 4%-12% NuPage Novex BIS-Tris SDS-polyacrylamide gels (Life Technologies, Burlington, ON, Canada) and electroblotted in MOPS transfer buffer to nitrocellulose membrane (Life Technologies, Burlington, ON, Canada). Rabbit polyclonal anti-HA (Abcam, Cambridge, England) or mouse monoclonal anti-beta-actin (abm, Richmond, BC, Canada) and Mouse TrueBlot ULTRA HRP-conjugated anti-mouse (Rockland Inc., Gilbertsville, PA, USA) or goat anti-rabbit IgG antibodies (Jackson ImmunoResearch Laboratories Inc., PA, USA) in 1∶5000 dilutions of 0.1% Tween-20, 5% bovine serum albumin (BSA), Tris-buffered saline (TBS) were used for protein detection. Proteins were visualised using Clarity Western enhanced chemiluminescence (ECL) Substrate (Bio-Rad, Hercules, CA, USA).

### ATRA cytotoxicity assay

To ensure that cells proliferated in vitro even if a MN1 mutation was non-functional, MN1 deletion constructs were transduced in bone marrow cells immortalized by retroviral expression of the fusion gene ND13. In vitro cytotoxicity assays were performed in Dulbecco's modified Eagle medium (DMEM) supplemented with 15% fetal bovine serum (FBS), 6 ng/mL murine interleukin 3 (mIL3), 10 ng/mL human interleukin 6 (hIL6), and 20 ng/mL murine stem-cell factor (mSCF; all from STEMCELL Technologies, Vancouver, BC, Canada). Cells were seeded at a cell density of 1×10^4^/mL in a 96 well plate, and incubated under light-protective conditions. ATRA (Sigma, Oakville, ON, Canada) was dissolved in DMSO (Sigma) and added to the culture medium at the specified concentrations as 1/1000^th^ of the final volume. After 64 hours, cells were stained with Alamar Blue (Sigma) for 8 hours and fluorescence was measured with a Tecan Safire^2^ microplate reader (Life Technologies, Burlington, ON, Canada). Viability was determined as percentage of DMSO-treated cells after background subtraction of fluorescence in wells with medium only. The 50% inhibitory concentration was determined as the concentration of ATRA that reduced cell viability to 50% of DMSO-treated cells.

### Mice and retroviral infection of primary bone marrow cells and bone marrow transplantation

Primary mouse bone marrow cells from 5-fluorouracil treated C57BL/6J donor mice (Faulding, Underdaler, Australia) were prestimulated for 48 hours, transduced by co-cultivation with viral producers for 48 hours, then harvested and plated into CFC media or directly transplanted into lethally irradiated syngeneic recipient mice, as previously described [32]. Recipient mice were exposed to a single dose of 750 to 810 cGy total-body irradiation accompanied by a life-sparing dose of 1×10^5^ freshly isolated bone marrow cells from syngeneic mice, and were monitored daily. Engraftment of transduced cells in peripheral blood was monitored every 4 weeks by fluorescence-activated cell-sorter (FACS) analysis and quantification of GFP-positive cells. Sick or moribund mice were sacrificed, spleens weighed, and red blood cells and white blood cells were counted using the scil Vet abc blood analyser (Vet Novations, Barrie, ON, Canada).

### Ethics Statement

C57BL/6J mice were bred and maintained in the Animal Research Centre of the British Columbia Cancer Agency as approved by the University of British Columbia Animal Care Committee (the Institutional Animal Care and Use Committee, IACUC). Experimental studies were approved by the University of British Columbia Animal Care Committe under experimental protocol numbers A04-0380 and A09-0009, and all efforts were made to minimise suffering.

### FACS analysis

Lineage distribution was determined by FACS analysis (FACSCalibur; Becton Dickinson, Mississauga, ON, Canada) as previously described [27]. Monoclonal antibodies used were phycoerythrin (PE)-labeled Gr1 (clone Ly6G-6C), B220 (CD45R), CD4, Ter119, and Sca-1 (Ly6A/E) and allophycocyanin (APC)-labeled CD11b, CD8, and c-kit (CD117) (BD Biosciences, Mississauga, ON, Canada).

### Bone marrow morphology

Cytospin preparations were stained with Wright-Giemsa stain. Images were visualised using a Nikon Eclipse 80i microscope (Nikon, Mississauga, ON, Canada) and a 20x/0.40 numerical aperture objective, or a 100x/1.25 numerical aperture objective and Nikon Immersion Oil (Nikon). A Nikon Coolpix 995 camera (Nikon) was used to capture images.

### Confocal microscopy

Twenty-four hours prior to fixation, micro growth glass cover slips (VWR International, Mississauga, ON, Canada) were coated in Cultrex Poly-L-Lysine (Trevigen, Gaithersburg, MD, USA). GP + E86 expressing cell lines expressing MN1, MN1Δ1, and MN1Δ5-7 were then plated. Cells were fixed in 4% paraformaldehyde in phosphate-buffered saline (PBS) for 10 minutes at room temperature, incubated with a 1∶500 dilution of rabbit anti-HA primary antibody followed by a 1∶300 dilution of anti-rabbit Alexa eFluor 594 secondary antibody (Life Technologies, Burlington, ON, Canada) and then stained with DAPI at 1 µg/mL (Sigma-Aldrich, St Louis, MO, USA). Slides were then mounted with DABCO mounting medium (Sigma-Aldrich, St Louis, MO, USA) and Z-stack photographs were taken 0.13 µm apart using a Leica TCS SP5 Confocal microscope (100x objective). Images were captured using LAS AF software (Leica Microsystems, Inc., Exton, PA, USA) and deconvoluted using Real-time GPU-based 3D Deconvolution [33] and DeconvolutionLab [34] in ImageJ.

### Gene expression profiling and gene set enrichment analysis

RNA was extracted using TRIZOL reagent (Life Technologies, Burlington, ON, Canada) from GFP+ cells that were sorted from mouse bone marrow cells four weeks after transplantation. Quality and integrity of the total RNA isolated was controlled by running all samples on an Agilent Technologies 2100 Bioanalyzer (Agilent Technologies, Mississauga, ON). Extracted RNA from MN1, MN1Δ1, and MN1Δ7 leukemia cells and Gr1+/CD11b+ bone marrow cells were hybridized to the Affymetrix GeneChip Mouse 430 2.0 (43.000 probes) microarray (n = 2) according to the manufacturer's instructions. Experiments were performed at the British Columbia Genome Sciences Centre, Vancouver, Canada. Gene expression can be found at the Gene Expression Omnibus database (GEO accession number GSE46990; http://www.ncbi.nlm.nih.gov/geo/query/acc.cgi?token=zhedbmoeuqcksli&acc=GSE46990). Data were analyzed using R and Bioconductor [35]. Quality was assessed with the ArrayQualityMetrics package [36]. Arrays were preprocessed using RMA [37]. Differentially expressed probesets were calculated with the LIMMA package [38] applying Benjamini-Hochberg multiple testing correction at an FDR of 0.05. For gene set enrichment the Broad Institute GSEA software package was used [39]. The datasets were collapsed into single genes and rank-ordered by signal to noise ratio. Gene set permutations were used to estimate statistical significance. Analyzed gene ontology sets were obtained from MSigDB v3.1 [39]. The gene set enrichment analysis software [39] (http://www.broad.mit.edu/gsea/index.jsp) was used to compare gene enrichment of Gene Ontology gene sets (dataset C5, available from the Molecular Signature database v3.1 [39]) between MN1Δ1 vs MN1 and MN1Δ7 vs. MN1.

### Statistical analysis

Comparisons were performed by unpaired T-tests. The two-sided level of significance was set at P less than 0.05. Comparison of survival curves were performed using the Kaplan-Meier method and log-rank test. Statistical analyses were performed with Excel (Microsoft Canada, Mississauga, ON, Canada) and GraphPad Prism 6 (GraphPad Software, La Jolla, CA, USA).

## Results

### The N-terminal region of MN1 is required for immortalization of bone marrow cells in vitro

**Figure 1:**
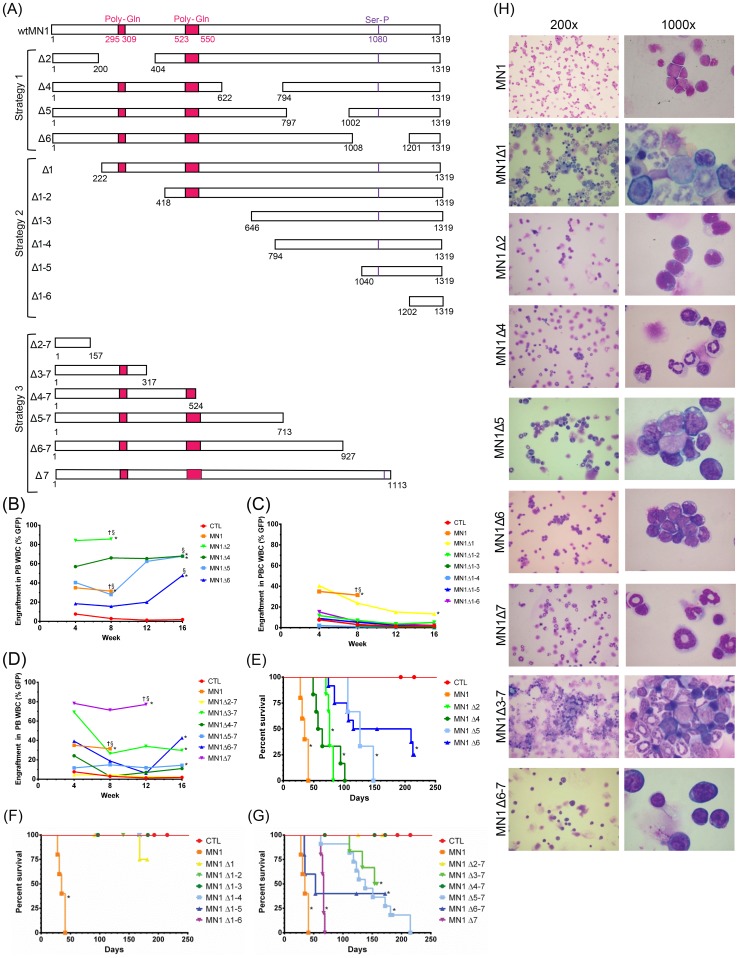
The N-terminal region of MN1 is required for its leukemogenic potential. (A) MN1 mutation constructs for structure-function analysis. In strategy 1 distinct stretches of approximately 200 amino acids were deleted throughout wildtype MN1. In strategy 2, stretches of approximately 200 amino acids were cumulatively deleted starting from the MN1 N-terminus. In strategy 3, stretches of approximately 200 amino acids were cumulatively deleted starting from the MN1 C-terminus. (B–D) Percentage of transgene-positive white blood cells engrafting in peripheral blood of transplanted mice at 4-week intervals. P values are given for the comparison of the indicated construct with CTL-transduced cells. The average engraftment is shown. Number of analysed mice and standard error can be found in Table S1. (E-G) Survival of mice receiving transplants of cells transduced with (E) strategy 1, (F) strategy 2, and (G) strategy 3 MN1 deletions. P values are given for the comparison of the indicated construct with CTL-transduced cells. The number of analysed mice is detailed in Table S1. (H) Morphology of bone marrow cells at death of diseased mice. The cells were Wright-Giemsa stained. Images were visualised using a Nikon Eclipse 80i microscope (Nikon, Mississauga, ON, Canada) and a 20x/0.40 numerical aperture objective, or a 100x/1.25 numerical aperture objective and Nikon Immersion Oil (Nikon). A Nikon Coolpix 995 camera (Nikon) was used to capture images. § engraftment in peripheral blood at the indicated time point or at death in cases where a mouse died before that time point. † all mice were dead at this time point due to disease. * indicates P<0.05, ** indicates P<0.001.

To elucidate the relationship between the structure of MN1 and the characteristics of MN1 leukemia, MN1 deletion mutants were generated in three strategies. Wildtype MN1 was divided into seven regions, each approximately 200 amino acids in length and numbered sequentially from the N-terminus. In an internal deletion series (strategy 1), distinct 200 amino acid regions were deleted (Figure 1A). Due to technical difficulties the construct lacking the third region from the N-terminus (MN1Δ3) did not express and was excluded from further analysis. Progressive N-terminal deletions (strategy 2) included six mutant constructs in which approximately 200 amino acid-regions were cumulatively deleted starting from the MN1 N-terminus. For progressive C-terminal deletions (strategy 3), stretches of approximately 200 amino acids were cumulatively deleted starting from the MN1 C-terminus (Figure 1A). The size and expression of all mutant constructs was validated at the RNA and protein level. The expected protein was detected for all constructs lacking one or two regions and for the constructs MN1Δ1-4, MN1Δ3-7, and MN1Δ5-7 lacking three or more regions. The remaining constructs lacking three or more regions did not, however, yield detectable protein (Figure S1A–B).

Freshly isolated bone marrow cells were transduced with MN1 mutation constructs or control (CTL) vector and plated in CFC medium, and replating ability and proportion of transduced cells (GFP+) were measured. While GFP-positive cells were lost in CTL-transduced cells after the second replating, GFP-positive cells outgrew non-transduced cells for internal deletion constructs (strategy 1) and could be replated up to the fifth plating, with the exception of MN1Δ6, where no colonies grew after the fourth plating (Figure S2A–B). For progressive N-terminal deletions (strategy 2), only cells lacking the most N-terminal domain (MN1Δ1) immortalized bone marrow cells in vitro, and competitively outgrew non-transduced cells; deletion of larger MN1 N-terminal stretches resulted in loss of replating capacity (Figure S2C and D). Cumulative deletions from the C-terminus (strategy 3) could immortalize bone marrow cells up to MN1Δ3-7, in which only 317 N-terminal amino acids are retained (Figure S2E and F), including MN1Δ4-7 which showed colony replating ability despite protein being unable to be detected by Western blot (Figure S1). In summary, the N-terminus of MN1 is necessary and sufficient to immortalize bone marrow cells in vitro with select regions, such as amino acids 1008–1201 playing a significant role in in vitro immortalization.

### The N-terminal domain of MN1 is required for its leukemogenic potential in vivo

MN1-transduced bone marrow cells were transplanted into lethally irradiated mice, and engraftment in peripheral blood was monitored monthly. Regardless of the mutation construct, mice showed at least minimal engraftment 4 weeks post-transplant (engraftment ≥1%). All internal deletion (strategy 1) constructs showed statistically significant higher engraftment than CTL mice with increasing engraftment over 16 weeks (Figure 1B). Progressive N-terminal deletion (strategy 2) constructs also showed engraftment at 4 weeks post-transplant, although engraftment levels did not significantly differ from control mice except for MN1Δ1, which showed higher early engraftment levels similar to full-length MN1. In addition, engraftment decreased over 16 weeks, suggesting that these constructs, including MN1Δ1, had defects in their proliferative and self-renewal capabilities and, thus, were unable to outcompete the co-transplanted normal bone marrow cells (Figure 1C and Table S3). Of the progressive C-terminal deletions (strategy 3), MN1Δ7 showed the highest engraftment levels, and MN1Δ6-7, MN1Δ5-7 and MN1Δ3-7 had significantly higher engraftment of transduced cells compared to CTL cells at 16 weeks (Figure 1D). The MN1 mutations that enhanced engraftment and proliferation in vivo also induced high WBC counts, anemia, and thrombocytopenia (Figures S3–S5, Tables S4–5).

All transplantation groups were fully characterized at time of sacrifice including bone marrow morphology with blast count, immunophenotype of GFP^+^ bone marrow cells, spleen weight and (for most constructs) secondary transplantations (Table S3). For mice succumbing to hematologic disease, the diagnosis is noted in Table S3 and supported by bone marrow morphology (Figure 1H). In summary, deletions including the first 221 N-terminal amino acids prevented MN1-induced AML, except one MN1Δ1 mouse that died with low engraftment, low WBC count, and a non-elevated blast count (Figure 1F). Confocal microscopy of cells expressing MN1Δ1 showed the protein present in both the cytoplasm and the nucleus to a similar extent as MN1, suggesting that loss of this region did not impact the ability of the mutant to localize to the nucleus. (Figure S6). Deletion of domains 2, 5, 6, or 7 did not affect the ability of MN1 to induce AML, although their loss significantly prolonged disease latency (Figure 1E). Deletion of domain 4 resulted in a rapid disease onset with low blast count, most likely a myelproliferative disease (Figure 1E and G). Combined deletion of domains 6 and 7 (MN1Δ6–7) at the C-terminus resulted in AML with 60% penetrance (Figure 1G). Interestingly, despite showing nuclear localization of the protein by confocal microscopy (Figure S6), deletion of domains 5–7 (MN1Δ5–7) at the C-terminus in two independent experiments resulted in T-lymphoblastic leukemia (see below). The minimal portion of MN1 with biologic function was MN1Δ3-7, corresponding to the 317 amino acids at the N-terminus, which induced a myeloproliferative disease with long latency and 50% penetrance (Figure 1G). In summary, these data suggest that the N-terminus of MN1 is required and sufficient for its proliferation-enhancing function in vivo (see also Table 1).

**Table 1:** Role of MN1 regions in leukemia cell fate regulation.

Phenotype	Required Domain(s)	Dispensable Domain(s)	Deletions likely too large to have any effect
**Proliferation/Self-Renewal**	1	One of: 3, 4, 5, 6, 7	2–7, 1–2, 1–3,1–4, 1–5, 1–6
**Myeloid Differentiation Block**	2, 7	One of: 1, 4, 5, 6	
**Megakaryocyte/Erythroid Differentiation Block**	1	One of: 2, 4, 5, 6, 7, 3–7, 5–7	
**ATRA resistance**	5, 6, 7	One of: 1, 2, 4	
**Lymphoid Differentiation Block**	5–7	One of: 1, 2, 4	

### The N-terminal region of MN1 is required to block megakaryocyte/erythroid differentiation

**Figure 2:**
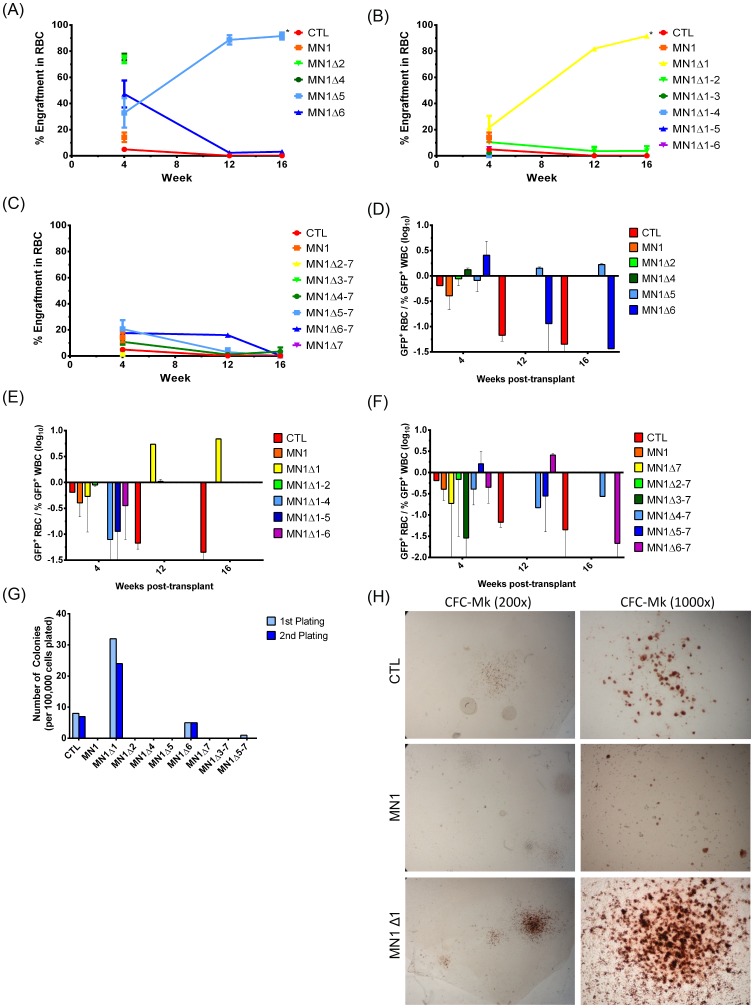
The N-terminal region of MN1 is required to block megakaryocyte/erythroid differentiation. (A–C) Percentage of transgene positive red blood cells engrafting in peripheral blood of transplanted mice at 4-week intervals. P values are calculated for the comparison of the indicated construct with CTL-transduced cells. The number of analysed mice and standard error is detailed in Table S3. (D–F) Proportion of red blood cells (RBC) compared to white blood cells (WBC) expressing (D) strategy 1, (E) strategy 2, or (F) strategy 3 MN1 deletion constructs after transplantation. P values are calculated for the comparison of the indicated construct with CTL-transduced cells. The number of analysed mice and standard error is detailed in Table S3. (G) Megakaryocyte colony-forming ability of mouse bone marrow cells transduced with MN1 deletion constructs (mean ± SD, n = 4). (H) Micrographs of representative CFC-Mk slides at the end of the first plating of bone marrow cells transduced with CTL vector, full-length MN1 or MN1Δ1. Images were visualised using a Nikon Eclipse 80i microscope (Nikon, Mississauga, ON, Canada) and a 20x/0.40 numerical aperture objective, or a 100x/1.25 numerical aperture objective and Nikon Immersion Oil (Nikon). A Nikon Coolpix 995 camera (Nikon) was used to capture images. * indicates P<0.05.

Peripheral blood from mice transplanted with MN1-transduced bone marrow cells was also assayed at 4-week intervals to determine the engraftment of red blood cells. The majority of mutants showed decreasing red blood cell engraftment over the 16-week period or the lifetime of the mouse. MN1Δ2 and MN1Δ4 mice had high engraftment levels at 4 weeks corresponding to high WBC engraftment. Only two constructs, MN1Δ1 and MN1Δ5, showed an increase in red blood cell engraftment over time, although the absolute number of red blood cells and hemoglobin did not increase in these mice (Figure 2A–C). When comparing the ratio of transgene positive RBC to WBC, MN1Δ1 and to a lesser extent MN1Δ5 were the only mutant constructs that showed a higher engraftment in red blood cells than white blood cells; a difference that increased over 16 weeks (Figure 2D–F). To assess the ability of MN1 deletion mutants to support megakaryocyte differentiation, we performed CFU-Mk assays of all internally-deleted (strategy 1) and select N- and C-terminally deleted (strategy 2 and 3) constructs. CTL cells, but not full-length MN1 cells, formed few, small CFU-Mk colonies. Similar to full-length MN1 cells, most MN1 deletion mutants were unable to form CFU-Mk colonies. However, N-terminally deleted (MN1Δ1) cells gave rise to 2 to 3-fold more colonies and larger colonies than control-transduced cells, sustained over two replatings (Figure 2G and H). A small number of colonies were also derived from MN1Δ6-transduced cells (Figure 2G). Together, these experiments suggest that the ability of MN1 to block erythroid-megakaryocyte differentiation can be localized to the N-terminus, with some contribution of domains 5 and 6 (see also Table 1).

### The C-terminal region of MN1 is required to block myeloid differentiation

**Figure 3:**
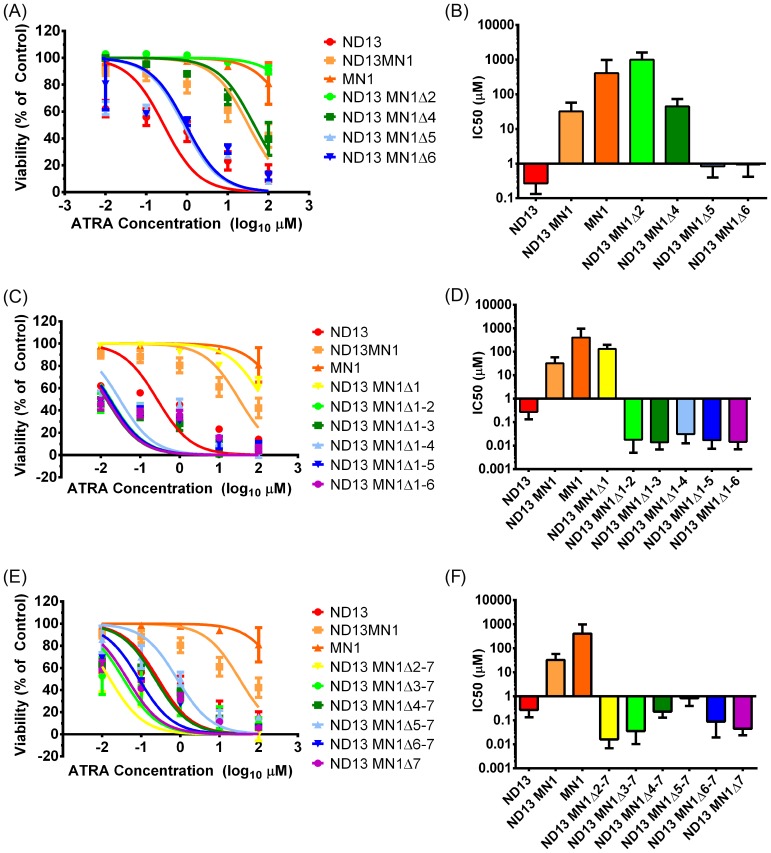
The C-terminal region of MN1 is required to block myeloid differentiation. (A–F) In vitro sensitivity to ATRA of ND13-immortalized cells that were transduced with MN1 deletion constructs. Dose-response curves are shown in the left panels (A, C, E) and IC50 values are shown in the right panels (B, D, F) for each deletion strategy (mean ± SD, n≥6).

To assess the effect of MN1 deletions on resistance to ATRA, we transduced ND13 bone marrow cells, previously reported to immortalize cells in vitro [27], with all MN1 deletion mutants. ND13 control cells were sensitive to *in vitro* ATRA administration with an IC50 of 0.27 µM. ND13+MN1-transduced cells were highly resistant with an IC50 of 32.4 µM, while MN1-transduced cells were even more ATRA resistant with an IC50 beyond 100 µM. When distinct regions were internally deleted from MN1 (strategy1), loss of domains 2 or 4 had no effect on ATRA resistance (Figure 3A). However, loss of domain 5, 6, or 7 restored ATRA sensitivity of the cells (Figure 3B). Progressive N-terminal deletions (strategy 2) with 2 or more domains deleted from the N-terminus were sensitive to ATRA (Figure 3C and D). All constructs with cumulative deletions of the C-terminus (strategy 3) were sensitive to ATRA (Figure 3E and F), highlighting the importance of the most C-terminal 206 amino acids of MN1 for ATRA resistance. These data suggest that the MN1 C-terminus plays an important role in regulating resistance to ATRA in MN1 cells, with the MN1 N-terminus (amino acids 222–418) being important for maintaining functionality of the MN1 protein.

**Figure 4:**
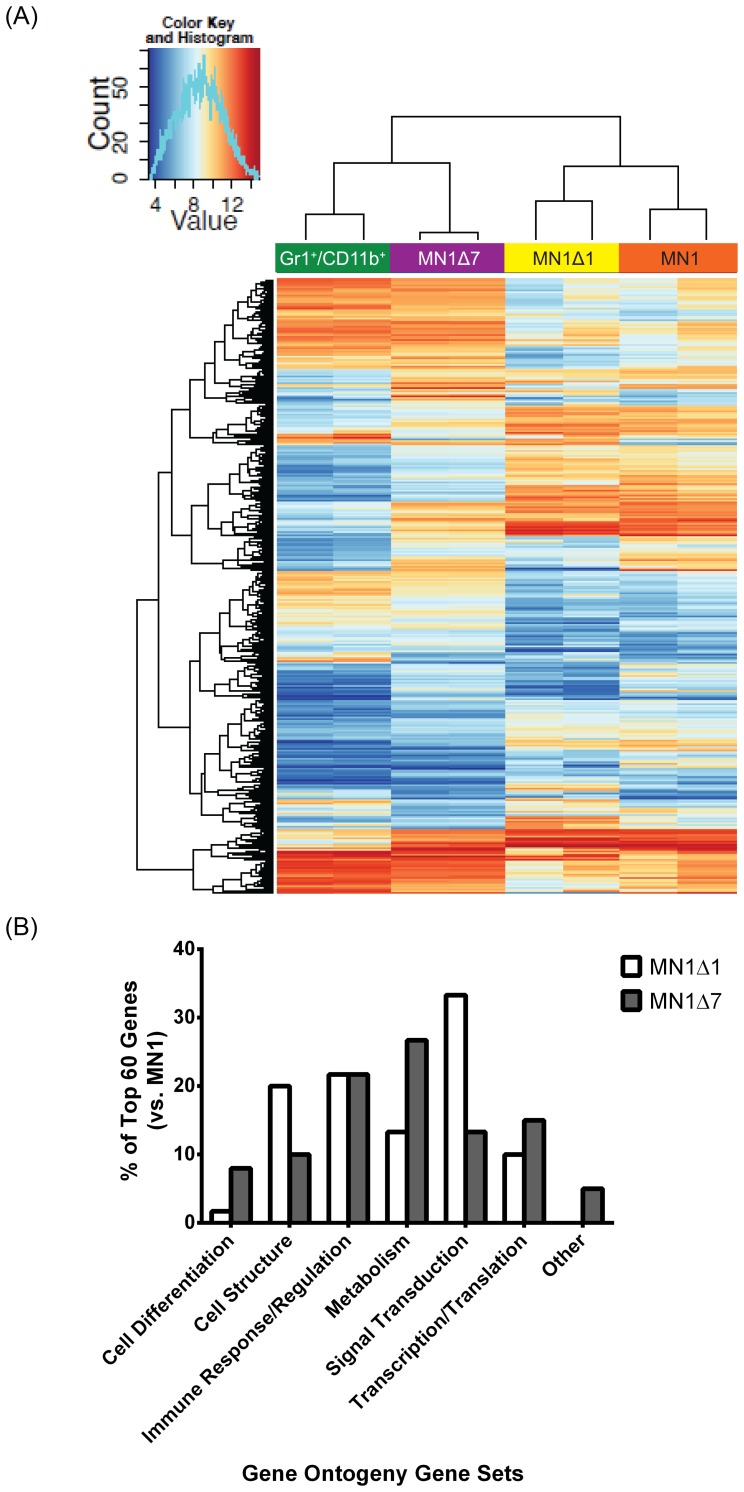
Hierarchical clustering of cells with N- and C-terminally deleted MN1. (A) Heat map of differentially regulated pathways for enhanced proliferation and blocked differentiation. (B) Comparison of top 60 enriched gene ontology gene sets for the comparison of MN1Δ1 and MN1Δ7 with wildtype MN1.

We performed gene expression profiling on GFP-positive bulk MN1-, MN1Δ1- and MN1Δ7-transduced bone marrow cells, and normal Gr1^+^CD11b^+^ differentiated myeloid cells sorted from bone marrow. Unsupervised hierarchical clustering showed that bulk C-terminally deleted MN1 cells (MN1Δ7, with an average 26.9% GFP^+^/Gr1^+^/CD11b^+^ population) clustered with Gr1^+^CD11b^+^ normal myeloid cells, which have been previously shown to have low Mn1 expression [17]. Alternatively, N-terminally deleted MN1 cells (MN1Δ1, with an average 24.3% GFP^+^/Gr1^+^/CD11b^+^ population) clustered with wildtype MN1 cells (Figure 4A). Comparison of the 60 most differentially expressed gene ontology gene sets between wildtype MN1 and MN1Δ1 or MN1Δ7 cells showed that those related to differentiation and metabolism were overrepresented in MN1Δ7 cells compared to MN1Δ1 cells (Figure 4B and Table S7). Conversely, gene sets related to signal transduction and cell structure were overrepresented in MN1Δ1 cells (Figure 4B and Table S8). The most differentially expressed genes in MN1Δ1 compared to MN1 cells were *HOXA9*, *HOXA10* and *MEIS2* (Table S9). These genes are among the most important genes driving self-renewal of HSCs, and their low expression in MN1Δ1 cells may explain their loss of leukemogenic potential. Several Krüppel-like factors were upregulated in MN1Δ1 cells, providing a link for their preferential erythroid differentiation. In MN1Δ7 cells 3 members of the eosinophil cationic protein (*Ecp* or *Ear1*, *2*, *3*) and eosinophil peroxidase were most differentially upregulated compared to MN1 cells (Table S10).

To compare the differentiation potential of cells transduced with different MN1 deletions, the immunophenotype of GFP positive cells in peripheral blood at week 4 post-transplant and in bone marrow at death was compared for all deletion constructs (Figures S7–S9). Expression of the progenitor cell marker cKit inversely correlated with those of the myeloid markers Gr1 and CD11b. Loss of the C-terminus and unexpectedly, also the loss of domain region 2, resulted in increased expression of myeloid markers Gr1 and CD11b, as well as mature neutrophils (MN1Δ7) and monocytic cells (MN1Δ2) besides blast cells in bone marrow smears of diseased mice (Figure 1H and Figures S7–S8). In summary, the C-terminal region is required to block myeloid differentiation and to induce resistance against ATRA, while domain 2 prevents myeloid differentiation but is dispensable for ATRA resistance (see also Table 1).

### A 606 amino-acid C-terminal region of MN1 is required to prevent T-lymphoid differentiation

**Figure 5:**
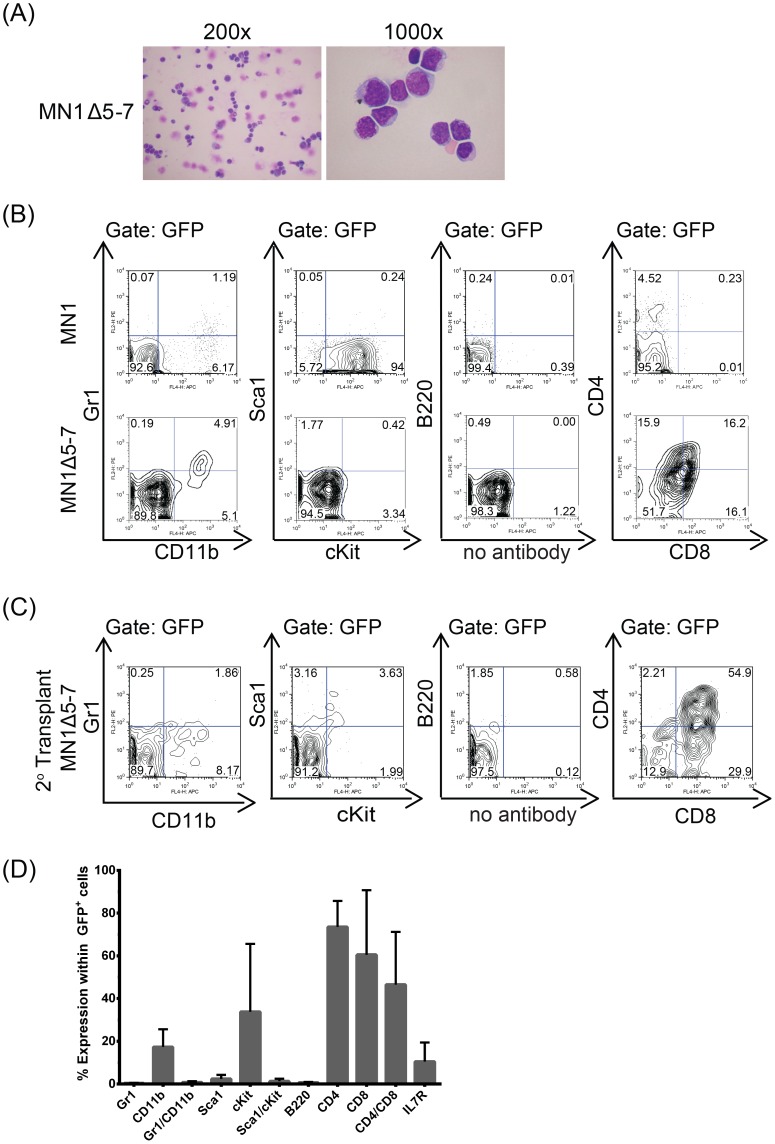
A 606 amino-acid C-terminal portion of MN1 prevents T-lymphoid differentiation. (A) Morphology of bone marrow cells from MN1Δ5-7 mice at death, showing a shift in leukemia from AML, as seen in MN1 leukemia, to an ALL leukemia upon loss of the C-terminal domains 5–7. (B) Representative immunophenotype of GFP^+^ MN1Δ5-7 bone marrow cells compared to wildtype MN1 bone marrow cells at death. (C) Representative immunophenotype of secondary transplants of GFP^+^ MN1Δ5-7 bone marrow cells at death. (D) Average cell surface marker expression for secondary transplants of GFP^+^ MN1Δ5-7 bone marrow cells at death (mean ± SEM, n = 3).

Combining deletion of the three most C-terminally located regions in MN1Δ5-7 resulted in delayed onset of leukemia with a median survival of 123 days (Figure 1G and Table S3). Interestingly, immunophenotypic analysis revealed CD4/CD8 double positive T cells within the GFP gate, and morphologic analysis showed blast cells in diseased mice in two independent experiments (Figure 5A–B), consistent with a diagnosis of T-lymphoblastic leukemia. Furthermore, these cells also induced T-ALL upon secondary transplantation (Figure 5C–D). Despite the differences in leukemic phenotype, MN1Δ5-7 was also shown to localise to the nucleus (Figure S6). In summary, these data suggest that the extended C-terminus of MN1 is required to block lymphoid differentiation and demonstrates the role of MN1 in regulating hematopoietic cell fate (see also Table 1 and Figure S9).

## Discussion

In this study, we systematically determined the functional properties of MN1 deletion mutants to identify regions that encode the key leukemogenic functions of MN1. Our analyses demonstrate that a single gene can induce leukemia by a “double-hit”, as the two functions promoting proliferation and inhibiting differentiation are encoded in structurally distinct regions. We also show that the myeloid or lymphoid lineage identity of leukemias can be determined by different mutations of the same oncogene, thus providing a potential explanation for the similar mutation spectrum in phenotypically distinct diseases like AML and T-ALL.

**Figure 6:**
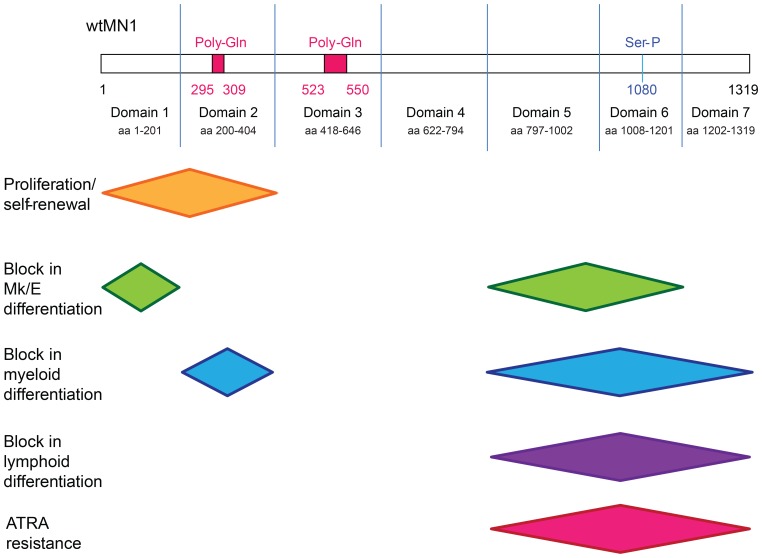
Functionally defined regions of MN1.

Deletion of the 221 most N-terminal amino acids (MN1Δ1) abolished the leukemogenicity of MN1 in vivo, as evidenced by decreased WBC engraftment in mice over time and failure to develop leukemia. However, the MN1Δ1 mutant provided both growth advantage and retention of ATRA resistance to bone marrow cells in vitro. We report the novel finding that MN1Δ1-transduced cells preferentially differentiated to the erythroid lineage in vivo and had an increased megakaryocyte differentiation potential in vitro, suggesting that the most N-terminal sequence of MN1 is also critical in blocking megakaryocyte/erythroid differentiation (Figure 6). Consistent with the reduced proliferative ability of MN1Δ1 cells, expression levels of HOXA9, HOXA10, and MEIS2 were most differentially downregulated compared to full-length MN1. In addition, JUN and FOS, factors of the AP1 complex, were most upregulated together with Kruppel-like factors (KLF) 2, 3, 4 and 6, which play an important role in erythroid differentiation and bind DNA at CACCC motifs [40]–[43]. Interestingly, the CACCC motif has also been identified as a consensus motif for MN1 binding to DNA in an oligonucleotide selection assay [44], [45].

Additional deletion of a region containing the first poly-Gln repeat (MN1Δ1-2) abolished any functional effect of MN1 in vitro and in vivo, despite the formation of protein. Conversely, the N-terminal sequence up to amino acid 317 (MN1Δ3-7) was sufficient to induce strong myeloproliferation with high WBC counts and large spleen with full myeloid differentiation potential, demonstrating that the MN1 N-terminus is driving proliferation in MN1 leukemia. We have previously shown that MN1 and MEIS1 share a high proportion of their regulatory chromatin sites and that MN1 leukemogenicity depends on MEIS1 [17]. Therefore, we speculate that the N-terminus is required for localization of MN1 to MEIS1 chromatin binding sites. In addition, van Wely *et al*. showed that MN1 interacts with P300 at amino acids 48 to 256, a region with considerable transcription activation function [44], the majority of which overlaps with domain 1. Future studies will be required to demonstrate if interaction between the MN1 N-terminus and P300 is required for the N-terminal functions promoting proliferation and blocking megakaryocyte/erythroid differentiation.

Several levels of evidence suggested that the MN1 C-terminus is required to inhibit myeloid differentiation (Table 1). First, loss of individual domains 5, 6, or 7 restored sensitivity to ATRA in vitro. Second, myeloid surface markers Gr1 and CD11b were most highly expressed in cells transduced with MN1Δ7 in vivo. In addition, gene expression profiling showed a close relationship of MN1Δ7 cells to differentiated myeloid cells, and more differentiation-related gene sets were upregulated in MN1Δ7 than in MN1Δ1 cells. Third, cumulative loss of domains 5, 6 and 7 resulted in loss of myeloid identity (see below). Lastly, cumulative deletion of domains 3 to 7 (MN1Δ3-7) resulted in a myeloproliferative disease with full differentiation potential to mature neutrophils (Table 1). These data suggest that the C-terminal regions (MN1Δ5, 6, 7) are the critical regions mediating resistance to ATRA-cytotoxicity, with some contribution from amino acids 222–418 (Figure 6). Recent work by Sharma et al. which showed that *Ccl9* and *Irf8* were upregulated in both MN1Δ7 cells and the MN1 model fused to the transcriptional activation domain VP16 (MN1VP16), suggesting that phenotypic similarities between the two models may be rooted in underlying gene expression patterns [46]. Van Wely et al. showed that MN1 binds to retinoic-acid response elements by an oligonucleotide selection assay [44] and, combined with our data, we hypothesize that the MN1 C-terminus directs MN1 to retinoic acid response elements to regulate transcription. Although retroviral overexpression, as used in this study, is likely to lead to artificially high transcriptional expression of MN1 and the mutants, we found that AML patients with the highest MN1 expression had similar expression levels to our murine MN1-transduced cell lines with the lowest MN1 expression, suggesting that at least some of our cell lines are comparable to patient data (data not shown). In addition, previous studies have shown that MN1 induces resistance to ATRA-induced differentiation and cell death [22], and that high-level expression of MN1 predicts ATRA resistance in AML patients, suggesting its future use as a biomarker for ATRA treatment [22], [47].

Deletion of a 606 amino acid fragment from the C-terminus reproducibly resulted in T-lymphoblastic leukemia with CD4- and CD8-double positive cells in mice. This suggests that the C-terminus of MN1 directs hematopoietic progenitor cells towards the myeloid lineage, but in its absence, allows differentiation towards the T cell lineage. Although this study cannot rule out that T-cell precursors may have been transduced by MN1Δ5-7, resulting in a bias or advantage towards lymphoid differentiation seen in the T-ALLs that developed, it is unlikely as findings were consistent in two independent experiments performed and supported by similar findings by an independent group [48]. Interestingly, mice lacking a portion of this 606 amino acid fragment, namely MN1Δ5 or MN1Δ7, developed AML. This suggests that amino acids 714–797 may be critical for myeloid differentiation, and it is only in their absence that lymphoid differentiation may occur. Kawagoe and Grosveld also described CD4/CD8 double positive T cell lymphomas in mice expressing the MN1/TEL fusion oncoprotein under the control of the AML1 promoter [49]. In this fusion protein, the 60 C-terminal amino acids of MN1 are lost due to the fusion to TEL [50]. As AML1 is also expressed in the T-lineage, these data suggest that overexpression of MN1 in T-progenitor cells can promote leukemogenesis, with Neumann and colleagues providing evidence of MN1 overexpression in T-lymphoblastic leukemias [51]. Taken together, we suggest that the C-terminus of MN1 encoded by amino acids 513–1319 (domains 5–7) instructs progenitor cells to the myeloid lineage and that in its absence progenitor cells can differentiate to the lymphoid lineage (Table 1).

During preparation of the present paper, one other group characterised functional MN1 regions, creating deletion constructs modelled off known MN1 protein domains in U937 cells [48]. Consistent with our data, Kandilci and colleagues reported decreased growth and colony-forming ability in vitro upon loss of the MN1 N-terminus. Kandilci *et al.* also showed that the loss of MN1 amino acids 570–1273 gave rise to T-cell lymphoma. This deleted region partially overlaps with our MN1Δ5-7 mutant, supporting the idea that the MN1 C-terminus promotes a myeloid-skew to MN1 leukemia. Finally, Kandilci *et al.* were also able to localise ATRA resistance to amino acids 18–458, but not 12–228, with their MN1-transduced U937 cells showing increased CD11b expression after 3 days of treatment. Interestingly, this region partially overlaps with domain 2 of our deletion mutants, providing support for our observation of increased CD11b expression in peripheral blood of animals transplanted with MN1Δ2. Kandilci *et al*. did not, however, report increased CD11b expression in C-terminal deletions, although this may be attributed to their most C-terminal deletion mutant retaining the 46 most C-terminal amino acids. It is possible that retention of these critical amino acids may have abrogated the differentiation effect seen in our complete deletions.

In summary, we characterized functional regions of the MN1 protein by a systematic mutation analysis and identified key regions that enhance proliferation and self-renewal, block myeloid, megakaryocytic/erythroid, and lymphoid differentiation, and induce resistance against ATRA. Our data support a critical function of MN1 as a key cell fate regulator in malignant hematopoiesis and provide a powerful new model for further dissection of the molecular events controlling transformation and the resulting leukemic phenotype.

## Supporting Information

Figure S1 **Expression levels of MN1 deletion constructs.** (A) Western blots illustrating the expression and size of protein products of the MN1 deletion constructs compared to full-length MN1. The figure is a composite of multiple gels with each lane representing a single construct stained with either anti-HA or anti-β-actin antibody. (B) Gel electrophoresis with PCR products illustrating the relative size of the MN1 deletion constructs compared to full-length MN1. (C–E) Expression levels of MN1 deletion constructs measured by qRT-PCR. MN1 deletion constructs were transduced in cells immortalized by NUP98HOXD13 (ND13). Mean ± SD, n = 3.
(TIF)

Figure S2 **Potential of MN1 variants to immortalize bone marrow cells in vitro.** Left panels (A, C, E) show number of CFC colonies per plating in methylcellulose under myeloid cytokine conditions. 5-FU pretreated bone marrow cells were transduced with MN1 deletions and were plated after transduction without sorting of cells. Right panels (B, D, F) show percentage of GFP positive cells at the end of each round of plating.
(TIF)

Figure S3 **White blood cell count in transplanted mice.** (A–C) White blood cell count (WBC) in peripheral blood of mice at 4-week intervals after transplantation. MN1 mutation constructs were used from (A) Strategy 1, (B) Strategy 2, and (C) Strategy 3. P values are given for the comparison of the indicated construct with CTL. The average WBC count is shown. Number of analyzed mice and standard error can be found in Table S5. § WBC count in peripheral blood at the indicated time point or at death in cases where a mouse died before that time point. † indicates that all mice were dead at this time point due to disease. * indicates P<0.05.
(TIF)

Figure S4 **Red blood cell count in transplanted mice.** (A–C) Red blood cell count (RBC) in peripheral blood of mice at 4 week intervals after transplantation. MN1 mutation constructs were used from (A) Strategy 1, (B) Strategy 2, and (C) Strategy 3. P values are given for the comparison of the indicated construct with CTL. The average RBC count is shown. Number of analyzed mice and standard error can be found in Table S5. § RBC count in peripheral blood at the indicated time point or at death in cases where a mouse died before that time point. † indicates that all mice were dead at this time point due to disease. * indicates P<0.05.
(TIF)

Figure S5 **Platelet count in transplanted mice.** (A–C) Platelet count in peripheral blood of mice at 4 week intervals after transplantation. MN1 mutation constructs were used from (A) Strategy 1, (B) Strategy 2, and (C) Strategy 3. P values are given for the comparison of the indicated construct with CTL. The average platelet count is shown. Number of analyzed mice and standard deviation can be found in Table S5. § Platelet count in peripheral blood at the indicated time point or at death in cases where a mouse died before that time point. † indicates that all mice were dead at this time point due to disease. * indicates P<0.05.
(TIF)

Figure S6 **Confocal microscopy of MN1-transduced cells.** Representative confocal microscopy images of GP + E86 cells transduced with (A) negative control, (B) MN1 tagged with an HA-tag, (C) MN1Δ1 with an HA-tag, and (D) MN1Δ5–7 with an HA-tag stained with (i) DAPI or (ii) anti-HA and (iii) DAPI and anti-HA merged.
(TIF)

Figure S7 **Immunophenotype of MN1-transduced cells in transplanted mice – stem and progenitor markers.** Percentage of GFP-expressing cells and expression of ckit and Sca1 in GFP+ cells in peripheral blood at 4 weeks and in bone marrow at death of mice receiving transplants of MN1-transduced cells. (A) Strategy 1, (B) Strategy 2, and (C) Strategy 3 MN1 constructs. Mean ± SEM. The number of analyzed mice is provided in Table S6.
(TIF)

Figure S8 **Immunophenotype of MN1-transduced cells in transplanted mice – myeloid markers.** Expression of myeloid markers in GFP+ cells in peripheral blood at 4 weeks and in bone marrow at death of mice receiving transplants of MN1-transduced cells. (A) Strategy 1, (B) Strategy 2, and (C) Strategy 3 MN1 constructs. Mean ± SEM. The number of analyzed mice is provided in Table S6.
(TIF)

Figure S9 **Immunophenotype of MN1-transduced cells in transplanted mice – T-cell markers.** Expression of T-cell markers in GFP+ cells in peripheral blood at 4 weeks and in bone marrow at death of mice receiving transplants of MN1-transduced cells. (A) Strategy 1, (B) Strategy 2, and (C) Strategy 3 MN1 constructs. Mean ± SEM. The number of analyzed mice is provided in Table S6.
(TIF)

Table S1 **MN1 deletion mutant primer sequences.**
(DOC)

Table S2 **MN1 qPCR primer sequences.**
(DOC)

Table S3 **Characterisation of mouse phenotype after transplantation with MN1 deletion constructs.**
(DOC)

Table S4 ***In vivo*** **engraftment of cells transduced with MN1 deletion constructs.**
(DOC)

Table S5 **Peripheral blood counts in mice receiving transplants of cells transduced with MN1 deletion constructs.**
(DOC)

Table S6 **Immunophenotype of GFP-positive cells in peripheral blood of mice receiving transplants of cells transduced with MN1 deletion constructs.**
(DOC)

Table S7 **Gene ontology gene sets enriched in MN1Δ7 cells compared to MN1 cells.**
(DOC)

Table S8 **Gene ontology gene sets enriched in MN1Δ1 cells compared to MN1 cells.**
(DOC)

Table S9 **Differentially Expressed Genes in MN1Δ1 cells compared to MN1 cells.**
(XLS)

Table S10 **Differentially Expressed Genes in MN1Δ7 cells compared to MN1 cells.**
(XLS)
