# Supplementary figures and images for: Cell Fate Decisions in Malignant Hematopoiesis: Leukemia Phenotype Is Determined by Distinct Functional Domains of the MN1 Oncogene
Source: PLoS One. 2014 Nov 17;9(11):e112671. doi: 10.1371/journal.pone.0112671 (PMC4234417; doi:10.1371/journal.pone.0112671)

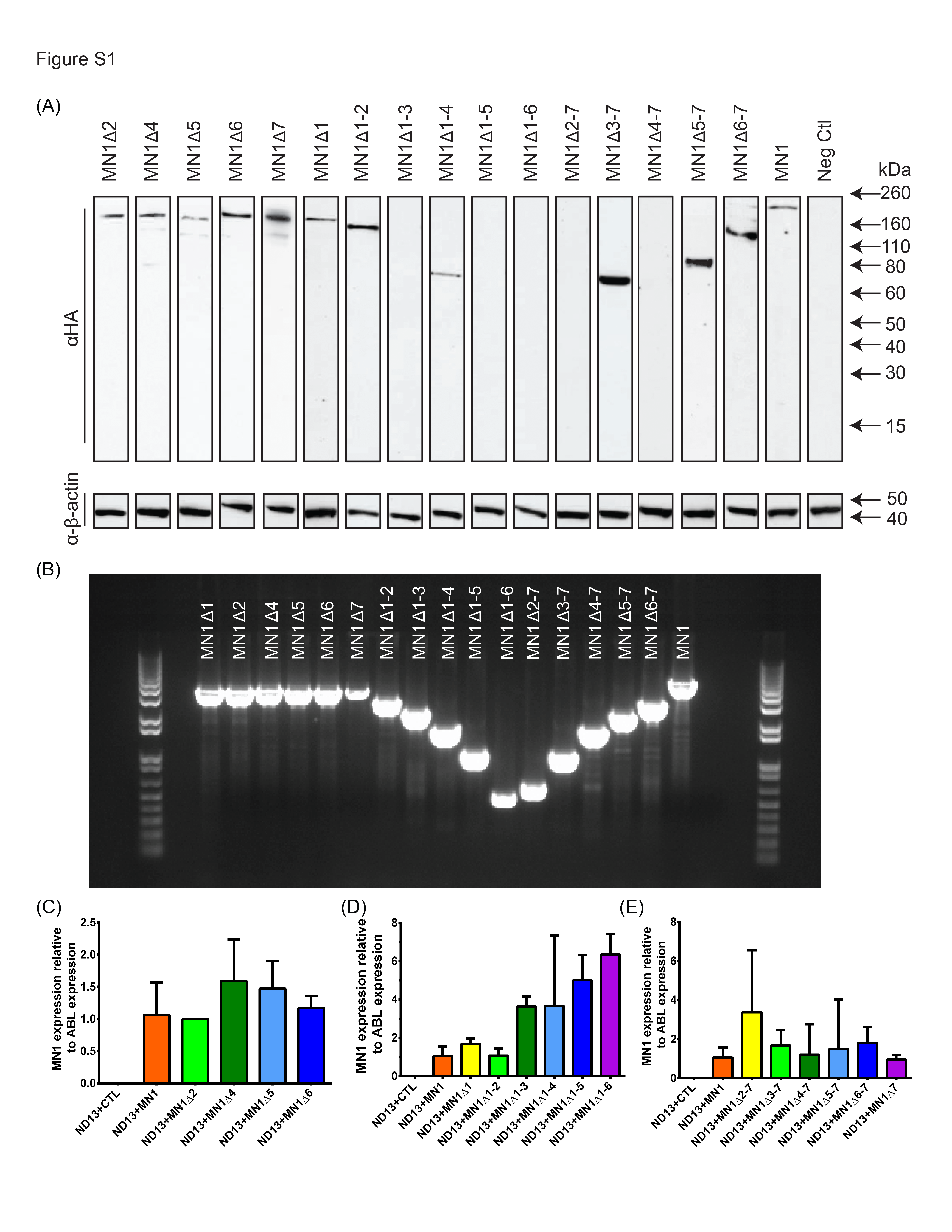

Supplement: Figure S1 — Expression levels of MN1 deletion constructs. (A) Western blots illustrating the expression and size of protein products of the MN1 deletion constructs compared to full-length MN1. The figure is a composite of multiple gels with each lane representing a single construct stained with either anti-HA or anti-β-actin antibody. (B) Gel electrophoresis with PCR products illustrating the relative size of the MN1 deletion constructs compared to full-length MN1. (C–E) Expression levels of MN1 deletion constructs measured by qRT-PCR. MN1 deletion constructs were transduced in cells immortalized by NUP98HOXD13 (ND13). Mean ± SD, n = 3. (TIF) [file pone.0112671.s001.tif]

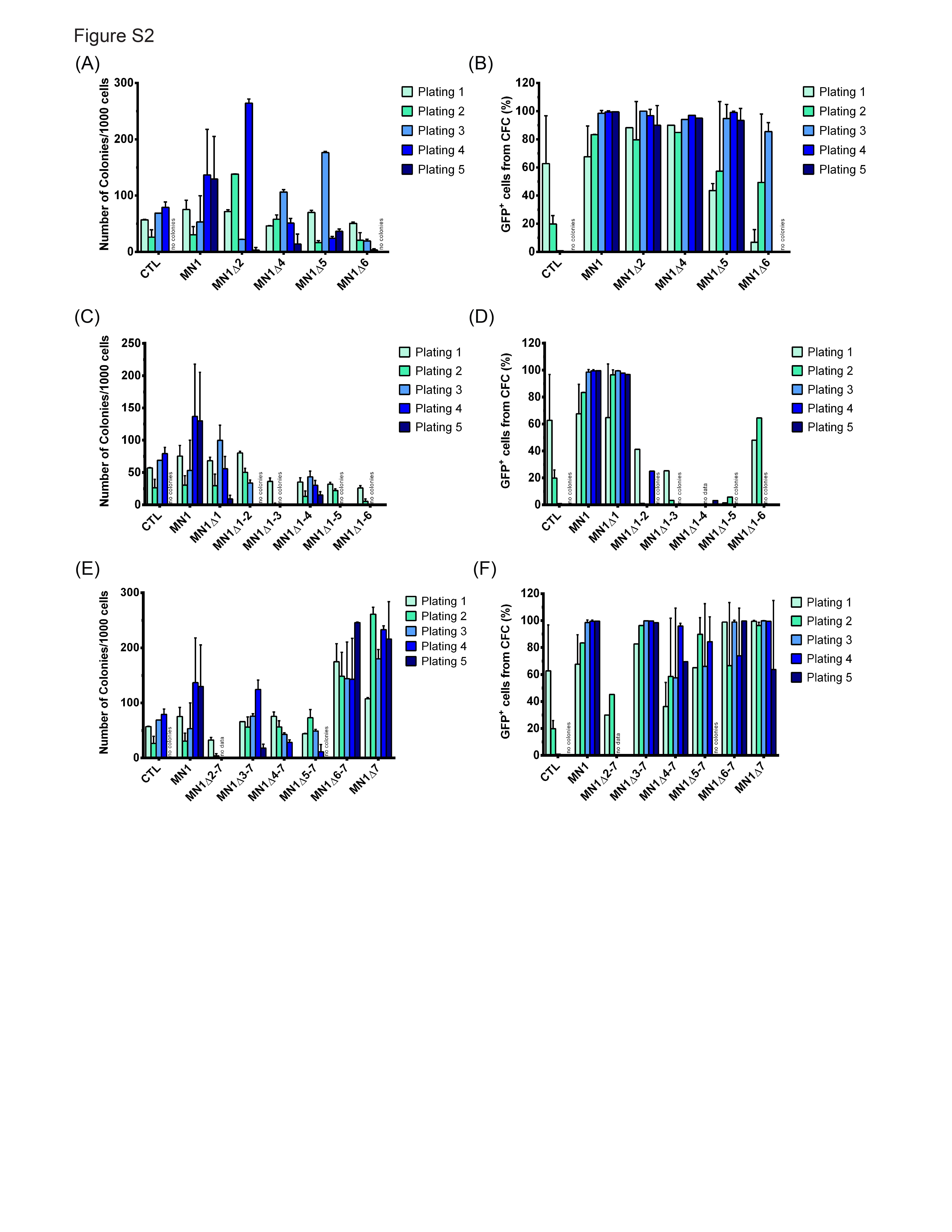

Supplement: Figure S2 — Potential of MN1 variants to immortalize bone marrow cells in vitro. Left panels (A, C, E) show number of CFC colonies per plating in methylcellulose under myeloid cytokine conditions. 5-FU pretreated bone marrow cells were transduced with MN1 deletions and were plated after transduction without sorting of cells. Right panels (B, D, F) show percentage of GFP positive cells at the end of each round of plating. (TIF) [file pone.0112671.s002.tif]

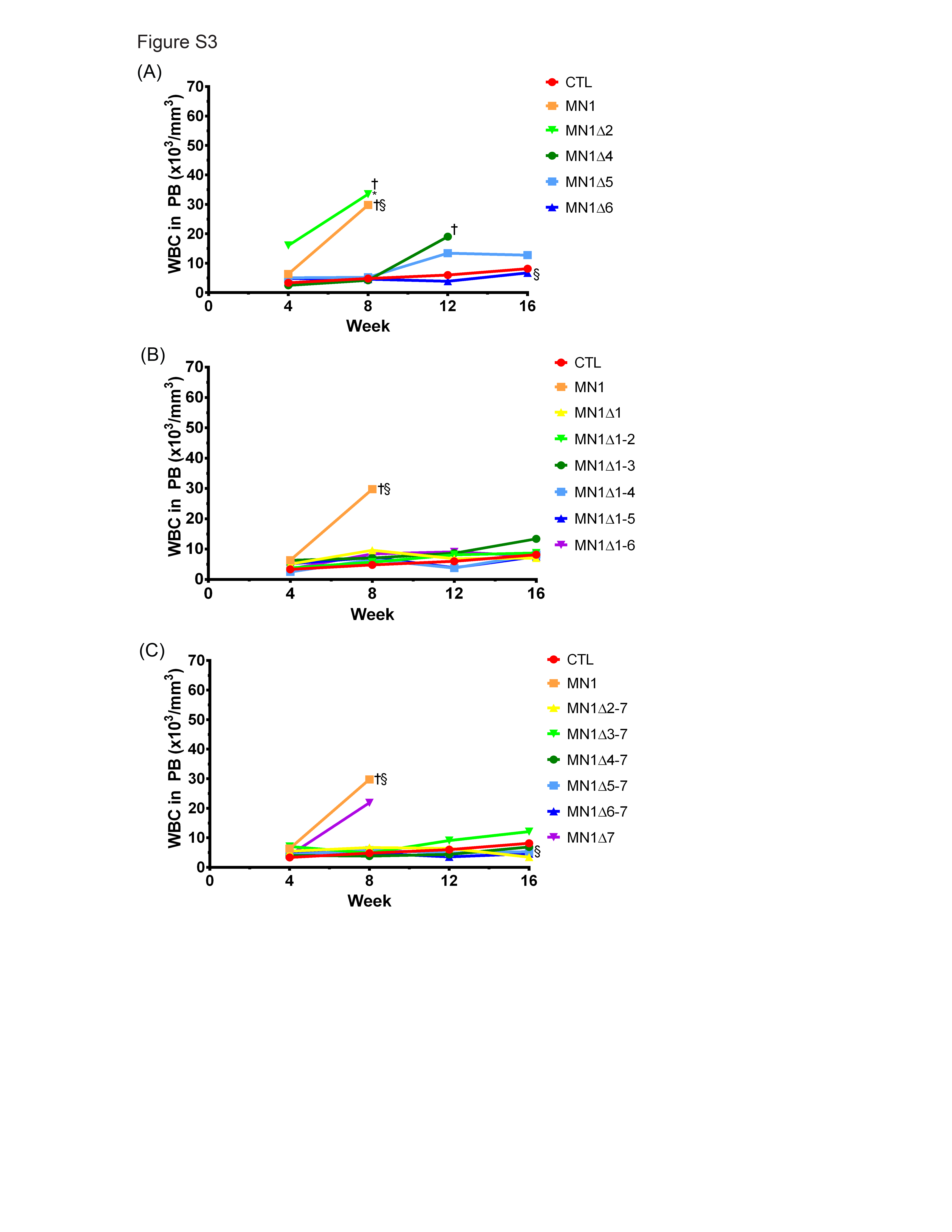

Supplement: Figure S3 — White blood cell count in transplanted mice. (A–C) White blood cell count (WBC) in peripheral blood of mice at 4-week intervals after transplantation. MN1 mutation constructs were used from (A) Strategy 1, (B) Strategy 2, and (C) Strategy 3. P values are given for the comparison of the indicated construct with CTL. The average WBC count is shown. Number of analyzed mice and standard error can be found in Table S5. § WBC count in peripheral blood at the indicated time point or at death in cases where a mouse died before that time point. † indicates that all mice were dead at this time point due to disease. * indicates P<0.05. (TIF) [file pone.0112671.s003.tif]

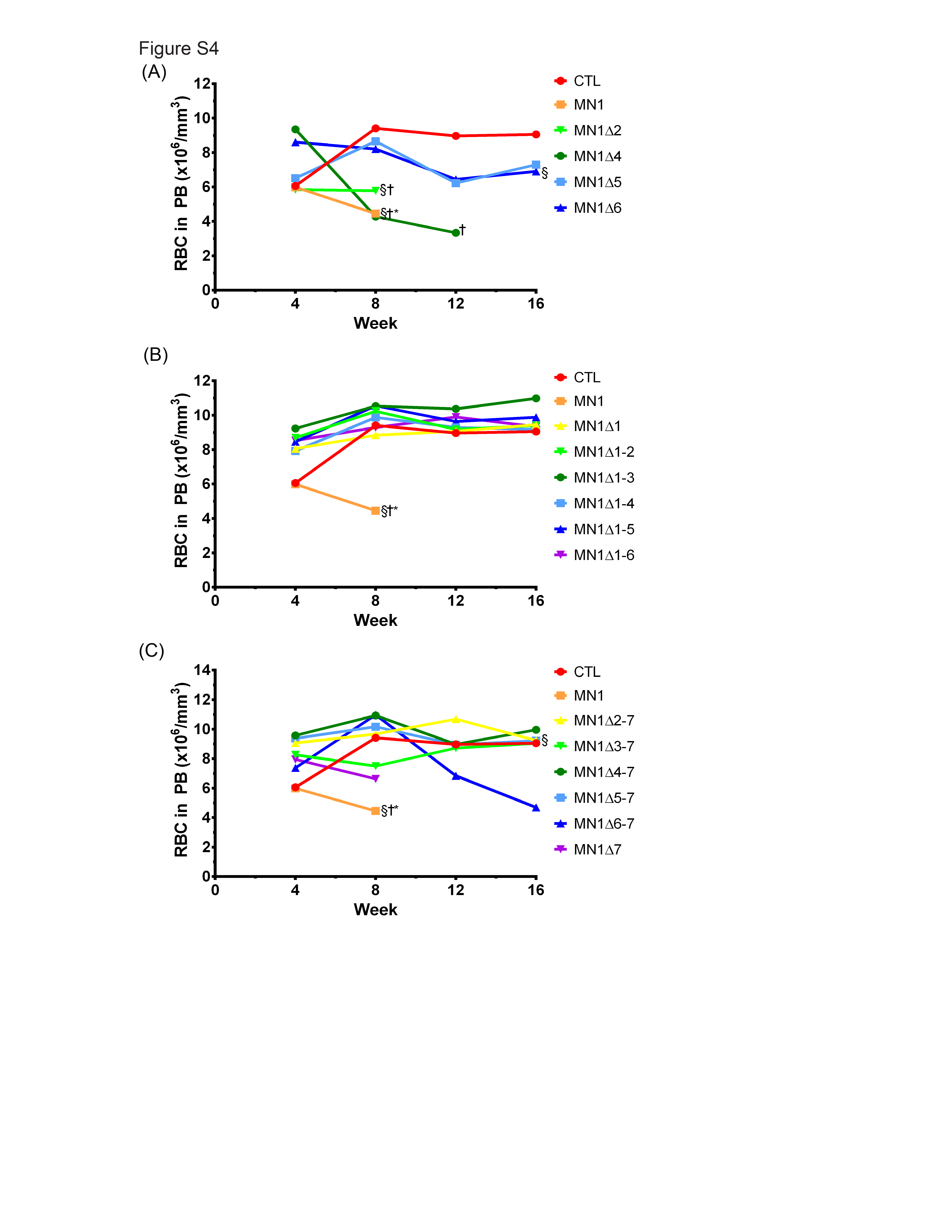

Supplement: Figure S4 — Red blood cell count in transplanted mice. (A–C) Red blood cell count (RBC) in peripheral blood of mice at 4 week intervals after transplantation. MN1 mutation constructs were used from (A) Strategy 1, (B) Strategy 2, and (C) Strategy 3. P values are given for the comparison of the indicated construct with CTL. The average RBC count is shown. Number of analyzed mice and standard error can be found in Table S5. § RBC count in peripheral blood at the indicated time point or at death in cases where a mouse died before that time point. † indicates that all mice were dead at this time point due to disease. * indicates P<0.05. (TIF) [file pone.0112671.s004.tif]

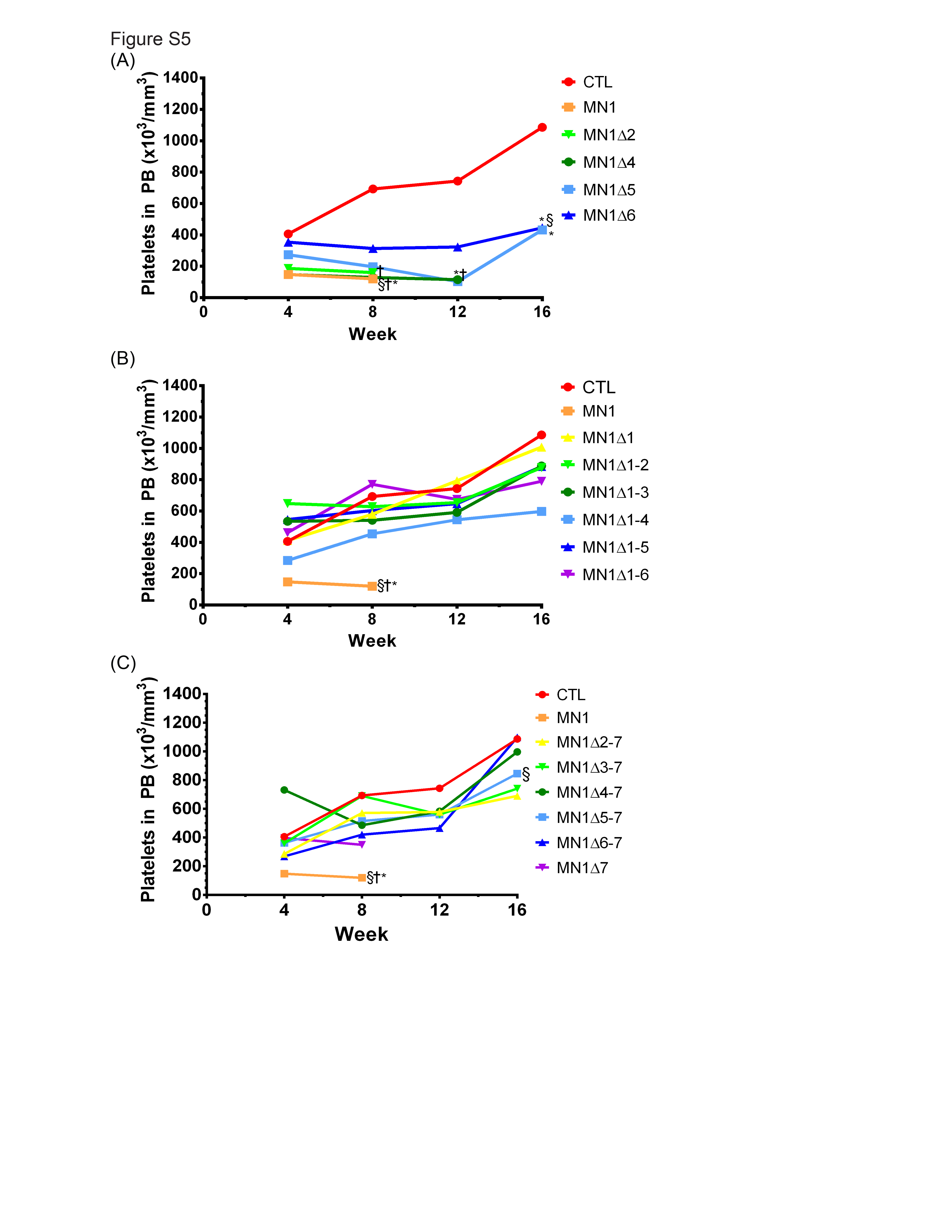

Supplement: Figure S5 — Platelet count in transplanted mice. (A–C) Platelet count in peripheral blood of mice at 4 week intervals after transplantation. MN1 mutation constructs were used from (A) Strategy 1, (B) Strategy 2, and (C) Strategy 3. P values are given for the comparison of the indicated construct with CTL. The average platelet count is shown. Number of analyzed mice and standard deviation can be found in Table S5. § Platelet count in peripheral blood at the indicated time point or at death in cases where a mouse died before that time point. † indicates that all mice were dead at this time point due to disease. * indicates P<0.05. (TIF) [file pone.0112671.s005.tif]

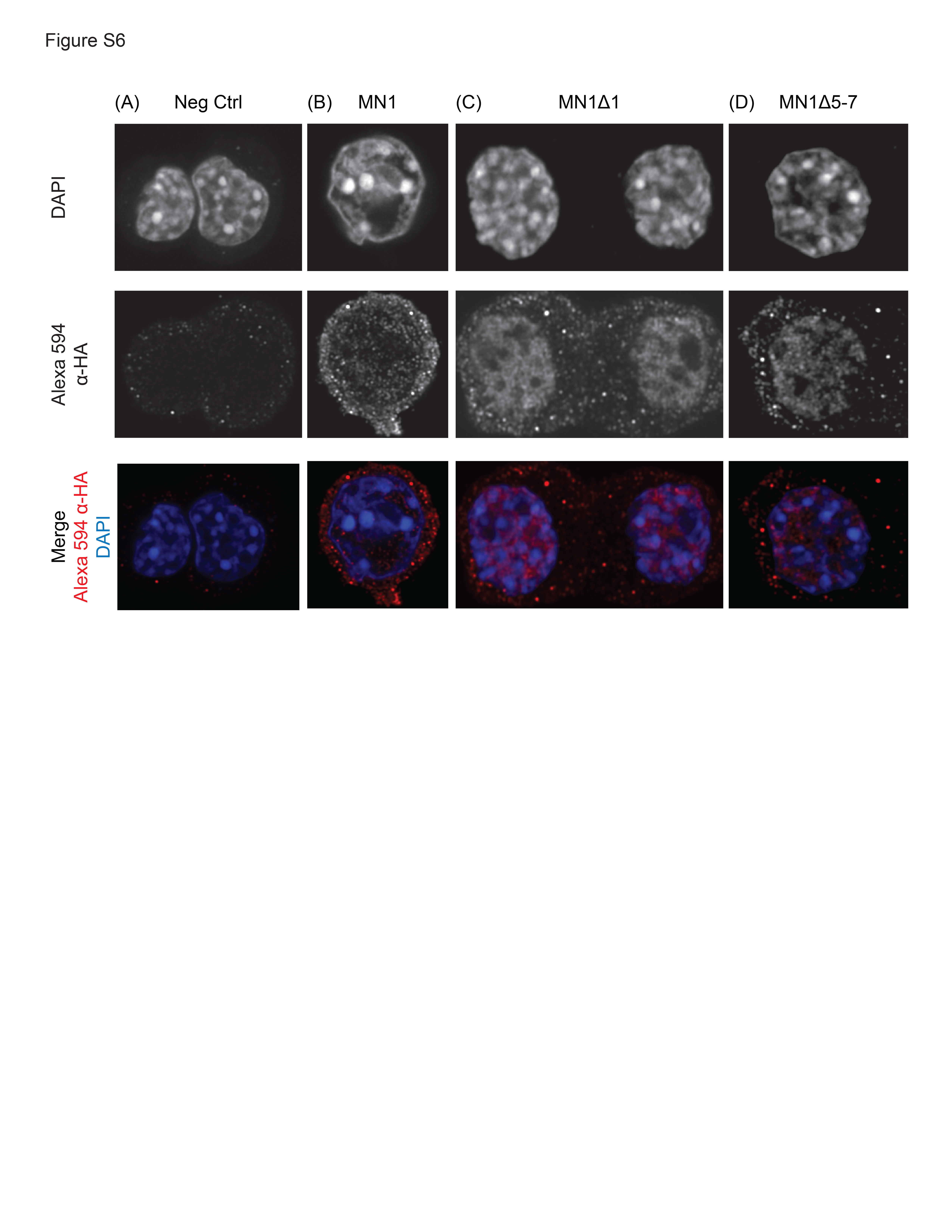

Supplement: Figure S6 — Confocal microscopy of MN1-transduced cells. Representative confocal microscopy images of GP + E86 cells transduced with (A) negative control, (B) MN1 tagged with an HA-tag, (C) MN1Δ1 with an HA-tag, and (D) MN1Δ5–7 with an HA-tag stained with (i) DAPI or (ii) anti-HA and (iii) DAPI and anti-HA merged. (TIF) [file pone.0112671.s006.tif]

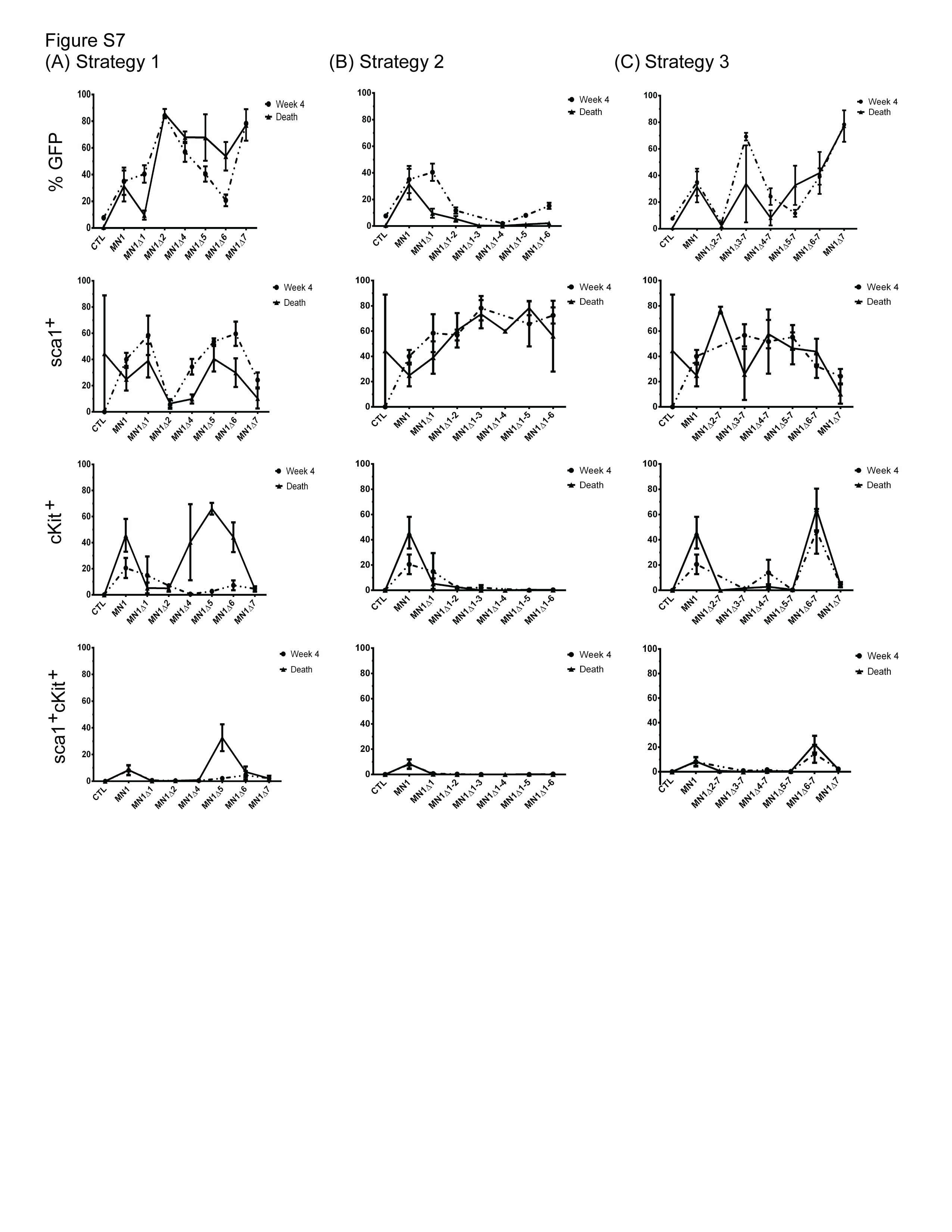

Supplement: Figure S7 — Immunophenotype of MN1-transduced cells in transplanted mice – stem and progenitor markers. Percentage of GFP-expressing cells and expression of ckit and Sca1 in GFP+ cells in peripheral blood at 4 weeks and in bone marrow at death of mice receiving transplants of MN1-transduced cells. (A) Strategy 1, (B) Strategy 2, and (C) Strategy 3 MN1 constructs. Mean ± SEM. The number of analyzed mice is provided in Table S6. (TIF) [file pone.0112671.s007.tif]

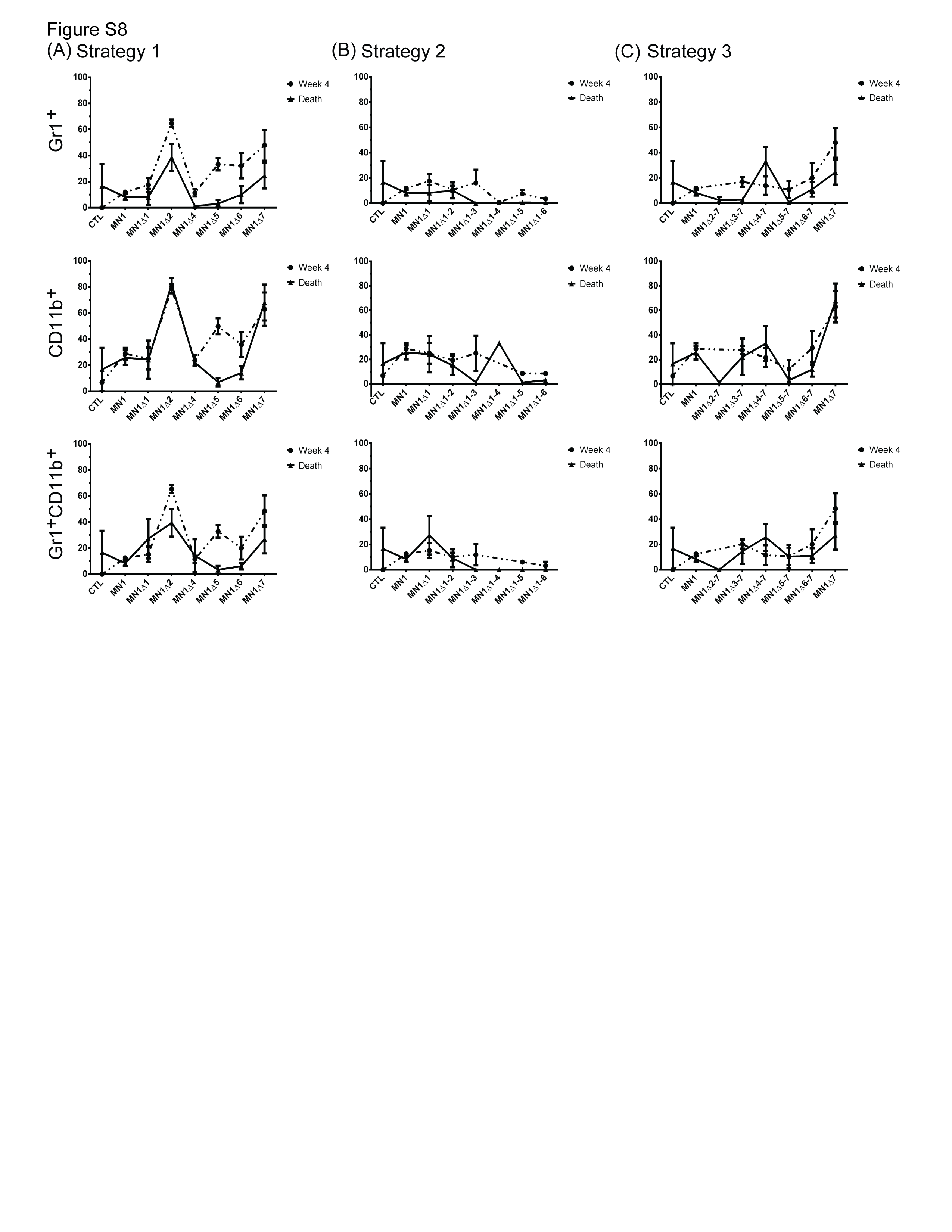

Supplement: Figure S8 — Immunophenotype of MN1-transduced cells in transplanted mice – myeloid markers. Expression of myeloid markers in GFP+ cells in peripheral blood at 4 weeks and in bone marrow at death of mice receiving transplants of MN1-transduced cells. (A) Strategy 1, (B) Strategy 2, and (C) Strategy 3 MN1 constructs. Mean ± SEM. The number of analyzed mice is provided in Table S6. (TIF) [file pone.0112671.s008.tif]

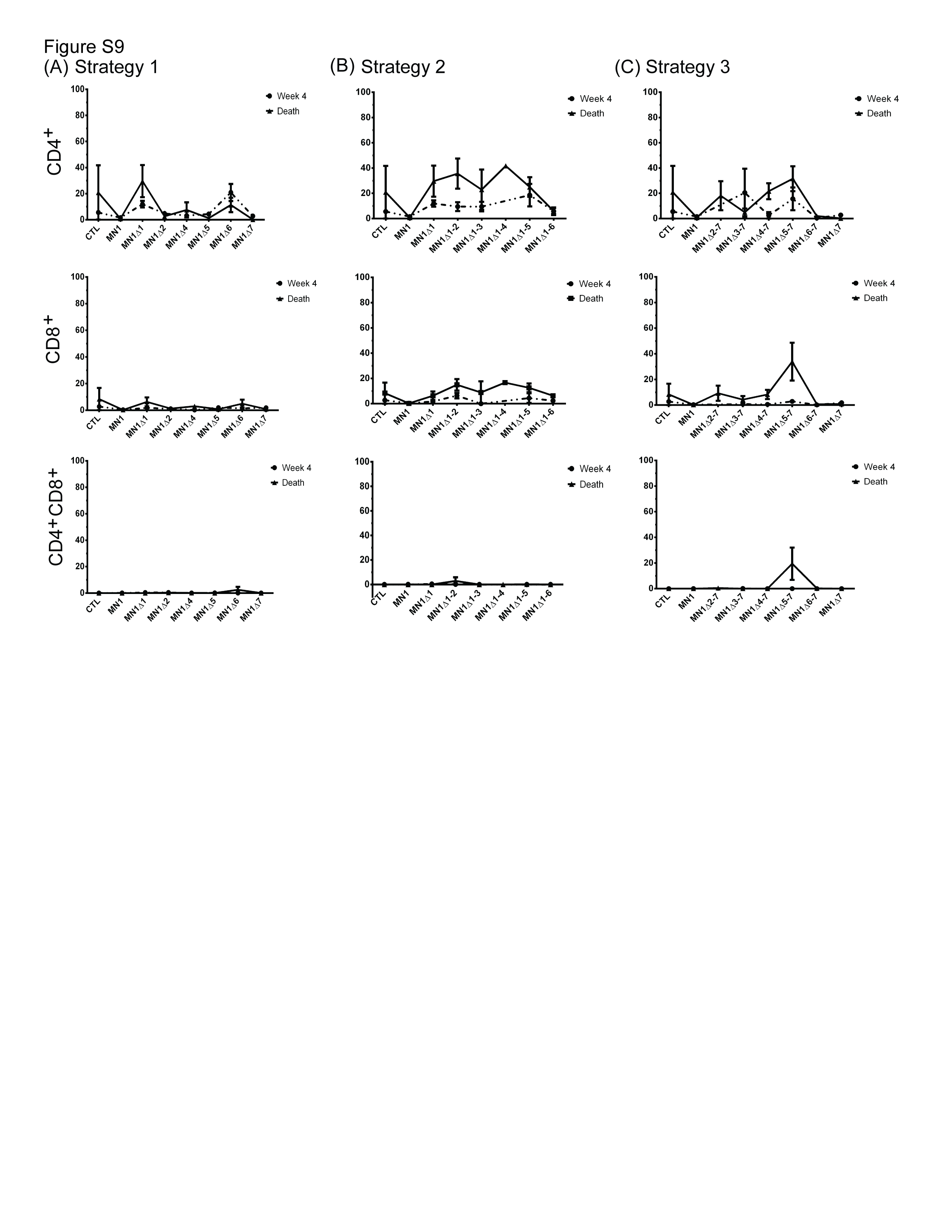

Supplement: Figure S9 — Immunophenotype of MN1-transduced cells in transplanted mice – T-cell markers. Expression of T-cell markers in GFP+ cells in peripheral blood at 4 weeks and in bone marrow at death of mice receiving transplants of MN1-transduced cells. (A) Strategy 1, (B) Strategy 2, and (C) Strategy 3 MN1 constructs. Mean ± SEM. The number of analyzed mice is provided in Table S6. (TIF) [file pone.0112671.s009.tif]
