# Supplementary material for: Cell Fate Decisions in Malignant Hematopoiesis: Leukemia Phenotype Is Determined by Distinct Functional Domains of the MN1 Oncogene
Source: PLoS One. 2014 Nov 17;9(11):e112671. doi: 10.1371/journal.pone.0112671 (PMC4234417; doi:10.1371/journal.pone.0112671)
Supplement: Table S1 — MN1 deletion mutant primer sequences. (DOC) [file pone.0112671.s010.doc]

**Supplementary Tables**

**Cell fate decisions in malignant hematopoiesis: Leukemia phenotype is determined by distinct functional domains of the MN1 oncogene**

Courteney K. Lai^1,2^, Yeonsook Moon^3^, Florian Kuchenbauer^4,5^, Daniel T. Starzcynowski^6^, Bob Argiropoulos^7^, Eric Yung^1^, Philip Beer^1^, Adrian Schwarzer^8^, Amit Sharma^8^, Gyeongsin Park^9^, Malina Leung^1^, Grace Lin^1^, Sarah Vollett^1^, Stephen Fung^1^, Connie J. Eaves^1,2^, Aly Karsan^10,11^, Andrew P. Weng^1,11^, R. Keith Humphries^1,2#^, Michael Heuser^12#^

**Table S1. MN1 de**letion mutant primer sequences

| **MN1 Strategy 1 Prox For** | TTTAAAGCGGCCGCATGTTTGGGCTGGAccaattc |
| --- | --- |
| **MN1 Strategy Dist Rev** | tttaaaGCGGCCGCTCA AGTTAGGGCAGCCACGAATG |
| **MN1Δ2 Prox For** | TTTAAAGCGGCCGCATGTTTGGGCTGGAccaattc |
| **MN1Δ2 Prox Rev** | TTTAAAAAGCTTGGCTCGGTTAGGGCTCTGGT |
| **MN1Δ2 Dist For** | TTTAAAAAGCTTGCGCAATTCGAGTATCCCATCCA |
| **MN1 Δ4 Dist Rev** | tttaaaaagcttcgcctgctgctcgaaggt |
| **MN1 Δ4 Prox Rev** | tttaaaaagcttcgcctgctgctcgaaggt |
| **MN1 Δ4 Dist For** | TTTAAAAAGCTTCAGCGCACCTCGGCCAGT |
| **MN1 Δ5 Prox Rev** | TTTAAAAAGCTTGGTGCGCTGGCTGGGCTG |
| **MN1Δ5 Dist For** | TTTAAAAAGCTTAAGGCGCTCACGTCGCCA |
| **MN1 Δ6 Prox Rev** | TTTAAAAAGCTTTGGCGACGTGAGCGCCT |
| **MN1 Δ6 Dist For** | TTTAAAAAGCTTTGCTGCTCCGAGGCGGTCA |
| **MN1 Strategy 2 Rev** | tttaaaGCGGCCGCTCA AGTTAGGGCAGCCACGAATG |
| **MN1Δ1 For** | TTTAAAGCGGCCGCATG TCCCACAGTCTGGAGCCA |
| **MN1 Δ1-2 For** | TTTAAAGzGGCCGCATGACGCGCAATTCGAGTATC |
| **MN1 Δ1-3 For** | TTTAAAGCGGCCGCATGCGaactttgagcgcgaAG |
| **MN1 Δ1-4 For** | TTTAAAGCGGCCGCATGTCCTTCAACAAGCCCAGCT |
| **MN1 Δ1-5 For** | TTTAAAGCGGCCGCATGGAAAAGGCGCTCACGTC |
| **MN1 Δ1-6 For** | TTTAAAGCGGCCGCATGTCCGAGGCGGTCAagaG |
| **MN1 Strategy 3 For** | TTTAAAGCGGCCGCATGTTTGGGCTGGAccaattc |
| **MN1 Δ7 Rev** | TTTAAAGCGGCCGCTCA GGTAGAGTTAGACATGATGC |
| **MN1 Δ2-7 Rev** | TTTAAAGCGGCCGCTCA GGATTCCAGGGTGTAGTTGG |
| **MN1 Δ3-7 Rev** | TTTAAAGCGGCCGCTCACTGCAGCTGACCCA |
| **MN1 Δ4-7 Rev** | TTTAAAGCGGCCGCTCACTGTTGCAGGGACTGGTG |
| **MN1 Δ5-7 Rev** | TTTAAAGCGGCCGCTCAGAACCTCTCAAAGAACAC |
| **MN1 Δ6-7 Rev** | tttaaaGcggccgCtcaCatgtgctcatagccct |
