# Supplementary material for: Cell Fate Decisions in Malignant Hematopoiesis: Leukemia Phenotype Is Determined by Distinct Functional Domains of the MN1 Oncogene
Source: PLoS One. 2014 Nov 17;9(11):e112671. doi: 10.1371/journal.pone.0112671 (PMC4234417; doi:10.1371/journal.pone.0112671)
Supplement: Table S2 — MN1 qPCR primer sequences. (DOC) [file pone.0112671.s011.doc]

**Supplementary Tables**

**Cell fate decisions in malignant hematopoiesis: Leukemia phenotype is determined by distinct functional domains of the MN1 oncogene**

Courteney K. Lai^1,2^, Yeonsook Moon^3^, Florian Kuchenbauer^4,5^, Daniel T. Starzcynowski^6^, Bob Argiropoulos^7^, Eric Yung^1^, Philip Beer^1^, Adrian Schwarzer^8^, Amit Sharma^8^, Gyeongsin Park^9^, Malina Leung^1^, Grace Lin^1^, Sarah Vollett^1^, Stephen Fung^1^, Connie J. Eaves^1,2^, Aly Karsan^10,11^, Andrew P. Weng^1,11^, R. Keith Humphries^1,2#^, Michael Heuser^12#^

Table S2. MN1 qPCR primer sequences

| MN1 qPCR N-term For | gtttgggctggaccaattc |
| --- | --- |
| MN1 qPCR N-term Rev | tgaacacccactttaaggcc |
| MN1 qPCR C-term For | cacttgcagtgcctgtctgt |
| MN1 qPCR C-term Rev | caacagatttgggacattcg |
