# Supplementary material for: Cell Fate Decisions in Malignant Hematopoiesis: Leukemia Phenotype Is Determined by Distinct Functional Domains of the MN1 Oncogene
Source: PLoS One. 2014 Nov 17;9(11):e112671. doi: 10.1371/journal.pone.0112671 (PMC4234417; doi:10.1371/journal.pone.0112671)
Supplement: Table S3 — Characterisation of mouse phenotype after transplantation with MN1 deletion constructs. (DOC) [file pone.0112671.s012.doc]

**Supplementary Tables**

**Cell fate decisions in malignant hematopoiesis: Leukemia phenotype is determined by distinct functional domains of the MN1 oncogene**

Courteney K. Lai^1,2^, Yeonsook Moon^3^, Florian Kuchenbauer^4,5^, Daniel T. Starzcynowski^6^, Bob Argiropoulos^7^, Eric Yung^1^, Philip Beer^1^, Adrian Schwarzer^8^, Amit Sharma^8^, Gyeongsin Park^9^, Malina Leung^1^, Grace Lin^1^, Sarah Vollett^1^, Stephen Fung^1^, Connie J. Eaves^1,2^, Aly Karsan^10,11^, Andrew P. Weng^1,11^, R. Keith Humphries^1,2#^, Michael Heuser^12#^

**Table S3. Characterisation of mouse phenotype after transplantation with MN1 deletion constructs.**

| **Construct** | | **CTL** | **MN1** | **MN1 Δ1** | **MN1 Δ2** | **MN1 Δ4** | **MN1 Δ5** | **MN1 Δ6** | **MN1 Δ7** | **MN1 Δ1-2** | **MN1 Δ1-3** | **MN1 Δ1-4** | **MN1 Δ1-5** | **MN1 Δ1-6** | **MN1 Δ2-7** | **MN1 Δ3-7** | **MN1 Δ4-7** | **MN1 Δ5-7** | **MN1 Δ6-7** |
| --- | --- | --- | --- | --- | --- | --- | --- | --- | --- | --- | --- | --- | --- | --- | --- | --- | --- | --- | --- |
| **No. of mice** | | 9 | 5 | 5 | 6 | 6 | 3 | 9 | 5 | 3 | 4 | 5 | 5 | 6 | 4 | 7 | 4 | 10 | 5 |
| **No. of mice dying from disease** | | 0 | 5 | 1 | 6 | 6 | 3 | 8 | 5 | 0 | 0 | 0 | 0 | 0 | 0 | 3 | 0 | 9 | 3 |
| **Engraftment in BM at death (% GFP)** | | 0.7 ± 0.5 (2) | 31.5 ± 11.6 (5) | 7.1 ± 3.0 (4) | 85. 7 ± 3.5 (3) | 68.0 ± 4.4 (5) | 67.8 ± 17.4 (3) | 53.7 ± 10.7 (9) | 77.2 ± 11.8 (5) | 3.5 ± 1.4 (3) | 0.4 ± 0.3 (3) | 0.2 ± 0.1 (5) | 1.3 ± 0.5 (5) | 2.2 ± 0.7 (3) | 2.2 ± 1.6 (2) | 33.8 ± 29.0 (3) | 8.2 ± 5.7 (4) | 38.4 ± 19.5 (6) | 42.0 ± 15.7 (5) |
| **WBC count at death (x10^3^/mm^3^)** | | 8.2 ± 1.8 (2) | 66.0 ± 44.0 (2) | 5.8 ± 0.5 (4) | 23.9 ± 3.1 (4) | 176.4 ± 94.4 (3) | 12.7 ± 4.9 (3) | 4.8 ± 1.0 (8) | 197.4 ± 73.8 (5) | n.d. | 8.9 ± 1.5 (2) | n.d. | n.d. | n.d. | n.d. | 127.6± 118.7 (3) | n.d. | 78.0 ± 43.4 (5) | 32.8 ± 9.7 (3) |
| **Hemoglobin count at death (g/dl)** | | 13.6 ± 1.4 (2) | 2.4 ± 0.2 (2) | 8.3 (1) | 6.6 ± 2.8 (4) | 0.0 (1) | 6.4 (1) | 2.2 ± 1.3 (4) | 7.8 ± 1.8 (5) | n.d. | 13.4 ± 0.4 (2) | n.d. | n.d. | n.d. | n.d. | 12.0 ± 1.4 (3) | n.d. | 8.5 ± 0.5 (5) | 2.4 ± 1.2 (3) |
| **Platelet count at death (x10^3^/mm^3^)** | | 1086.0 ± 138.0 (2) | 82.0 ± 9.0 (2) | 993.0 (1) | 119.3 ± 53.7 (4) | 31.0 (1) | 115.0 (1) | 27.3 ± 1.3 (4) | 280.8 ± 106.3 (5) | n.d. | 633.0 ± 68.0 (2) | n.d. | n.d. | n.d. | n.d. | 554.0 ± 222.7 (3) | n.d. | 317.9 ± 157.0 (5) | 76.7 ± 23.1 (3) |
| **Median survival time for diseased mice (days)** | | N/A | 35 (5) | 168 (1) | 76 (6) | 60.5 (6) | 126 (3) | 106 (8) | 67 (5) | N/A | N/A | N/A | N/A | N/A | N/A | 133 (3) | N/A | 123 (9) | 35 (3) |
| **Median observation time for mice not dying from disease** | | 268 (9) | N/A | 181 (3) | N/A | N/A | N/A | 154 (1) | N/A | 140 (3) | 139.5 (2) | 167 (5) | 168 (5) | 156.5 (6) | 146.5 (4) | 158 (4) | 154 (4) | 184 (1) | 147.5 (2) |
| **Blast %** | | n.d. | 57 ± 7 (5) | 12 ± 3 (5) | 39 ± 11 (3) | 6 ± 5 (2) | 60 ± 9 (2) | 92 ± 2 (4) | 50 ± 15 (3) | n.d. | n.d. | n.d. | n.d. | n.d. | n.d. | 1 ± 0 (2) | n.d. | 60 ± 13.5 (2) | 85 ± 9 (3) |
| **Construct** | | **CTL** | **MN1** | **MN1 Δ1** | **MN1 Δ2** | **MN1 Δ4** | **MN1 Δ5** | **MN1 Δ6** | **MN1 Δ7** | **MN1 Δ1-2** | **MN1 Δ1-3** | **MN1 Δ1-4** | **MN1 Δ1-5** | **MN1 Δ1-6** | **MN1 Δ2-7** | **MN1 Δ3-7** | **MN1 Δ4-7** | **MN1 Δ5-7** | **MN1 Δ6-7** |
| **% Gr1^+^ (BM)** | | 16.7 ± 16.7 (2) | 7.3 ± 2.4 (5) | 2.2 ± 2.2 (3) | 38.5 ± 10.6 (3) | 1.1 ± 0.6 (2) | 3.1 ± 2.9 (3) | 11.3 ± 7.3 (8) | 24.5 ± 9.7 (5) | 3.9 ± 1.2 (3) | 0.0 ± 0.0 (2) | 0.4 ± 0.4 (2) | 0.7 ± 0.4 (5) | 0.5 ± 0.3 (3) | 2.4 ± 2.4 (2) | 2.7 ± 1.7 (3) | 33.0 ± 11.4 (4) | 1.1 ± 1.0 (5) | 11.1 ± 5.9 (5) |
| **% CD11b^+^ (BM)** | | 16.7 ± 16.7 (2) | 26.1 ± 6.9 (5) | 13.0 ± 12.2 (3) | 82.2 ± 4.5 (3) | 22.1 ± 1.3 (2) | 6.9 ± 3.2 (3) | 15.9 ± 5.4 (8) | 68.0 ± 13.8 (5) | 10.1± 6.3 (3) | 0.0 ± 0.0 (2) | 33.3 (1) | 1.2 ± 0.5 (5) | 3.0 ± 0.9 (3) | 1.5 ± 1.5 (2) | 22.4 ± 14.8 (3) | 34.2 ± 13.4 (4) | 4.4 ± 2.7(5) | 12.1 ± 5.8 (5) |
| **% Gr1^+^CD11b^+^ (BM)** | | 16.7 ± 16.7 (2) | 8.5 ± 2.8 (5) | 24.3 ± 19.2 (3) | 39.4 ± 10.6 (3) | 14.3 ± 12.5 (2) | 3.4 ± 3.00 (3) | 6.8 ± 2.7 (8) | 26.9 ± 10.9 (5) | 2.0 ± 1.3 (3) | 0.0 ± 0.0 (2) | 0.0 (1) | 0.1 ± 0.1 (5) | 0.1 ± 0.1 (3) | 0.0 ± 0.0 (2) | 14.6 ± 10.0 (3) | 31.6 ± 11.9 (4) | 14.0 ± 12.1 (5) | 11.1 ± 5.9 (5) |
| **% cKit^+^ (BM)** | | 0.0 ± 0.0 (2) | 45.6 ± 12.6 (5) | 5.4 ± 5.4 (3) | 5.0 ± 2.6 (3) | 40.3 ± 29.2 (2) | 66.0 ± 4.5 (3) | 49.7 ± 11.3 (8) | 4.5 ± 2.1 (5) | 3.3 ± 2.3 (3) | 0.0 ± 0.0 (2) | 0.0 (1) | 0.5 ± 0.4 (5) | 0.3 ± 0.3 (3) | 0.0 ± 0.0 (2) | 1.7 ± 1.1 (3) | 2.8 ± 2.0 (3) | 0.6 ± 0.3 (5) | 64.0 ± 16.5 (5) |
| **% sca1^+^ (BM)** | | 44.45 ± 44.45 (2) | 24.86 ± 8.61 (5) | 44.10 ± 15.23 (3) | 6.35 ± 3.25 (3) | 9.84 ± 3.47 (2) | 40.50 ± 9.75 (3) | 31.08 ± 10.23 (8) | 10.2 ± 7.6 (5) | 72.9± 8.4 (3) | 62.5 ± 4.2 (2) | 60.0 (1) | 78.2 ± 5.7 (5) | 56.0 ± 28.0 (3) | 76.6 ± 2.7 (2) | 25.7 ± 20.2 (3) | 55.6 ± 15.7 (3) | 38.9 ± 15.8 (5) | 43.8 ± 10.2 (5) |
| **% cKit^+^sca1^+^ (BM)** | | 0.0 ± 0.0 (2) | 8.4 ± 3.6 (5) | 0.2 ± 0.2 (3) | 0.4 ± 0.2 (3) | 0.9 ± 0.0 (2) | 32.6 ± 10.0 (3) | 7.9 ± 4.4 (8) | 2.1 ± 2.0 (5) | 0.0 ± 0.0 (3) | 0.0 ± 0.0 (2) | 0.0 (1) | 0.2 ± 0.2 (5) | 0.0 ± 0.0 (3) | 0.7 ± 0.7 (2) | 0.1 ± 0.1 (3) | 0.6 ± 0.6 (3) | 0.6 ± 0.4 (5) | 22.5 ± 6.9 (5) |
| **% CD4^+^ (BM)** | | 20.9 ± 20.9 (2) | 0.8 ± 0.3 (5) | 35.7 ± 13.8 (3) | 2.6 ± 1.1 (3) | 7.5 ± 5.8 (2) | 1.2 ± 0.7 (3) | 6.1 ± 2.1 (8) | 0.2 ± 0.1 (5) | 43.3± 12.9 (3) | 26.8 ± 26.8 (2) | 41.7 (1) | 25.0 ± 7.8 (5) | 6.00 ± 2.4 (3) | 18.1 ± 11.4 (2) | 4.9 ± 3.1 (3) | 19.4 ± 8.3 (3) | 36.8 ± 9.9 (5) | 2.0 ± 1.5 (5) |
| **% CD8^+^ (BM)** | | 8.4 ± 8.4 (2) | 0.3 ± 0.2 (5) | 7.3 ± 4.1 (3) | 1.2 ± 0.5 (3) | 3.0 ± 0.9 (2) | 0.8 ± 0.4 (3) | 4.9 ± 3.0 (8) | 0.8 ± 0.5 (5) | 16.9± 5.9 (3) | 13.3 ± 13.3 (2) | 16.7 (1) | 12.6 ± 3.4 (5) | 6.3 ± 1.4 (3) | 9.3 ± 5.9 (2) | 4.3 ± 2.8 (3) | 11.1 ± 3.3 (3) | 39.6 ± 16.1 (5) | 0.6 ± 0.5 (5) |
| **% CD4^+^CD8^+^ (BM)** | | 0.00 ± 0.00 (2) | 0.03 ± 0.03 (5) | 0.00 ± 0.00 (3) | 0.29 ± 0.23 (3) | 0.09 ± 0.09 (2) | 0.07 ± 0.06 (3) | 0.19 ± 0.13 (8) | 0.01 ± 0.01 (5) | 3.90 ± 3.90 (3) | 0.00 ± 0.00 (2) | 0.00 (1) | 0.20 ± 0.20 (5) | 0.00 ± 0.00 (3) | 0.48 ± 0.48 (2) | 0.02 ± 0.02 (3) | 0.00 ± 0.00 (3) | 22.70 ± 14.42 (5) | 0.23 ± 0.22 (5) |
| **Spleen weight at death (g)** | | 0.06 ± 0.02 | 0.47 ± 0.02 (2) | n.d. | 0.67 ± 0.41 (2) | 1.00 ± 0.87 (2) | 0.73 ± 0.10 (2) | 0.20 ± 0.06 (7) | 0.32 ± 0.08 (3) | n.d. | n.d. | n.d. | n.d. | n.d. | n.d. | 0.48 (1) | n.d. | 0.50 ± 0.22 (3) | 0.31 ± 0.07 (3) |
| **Diagnosis** | | n.d. | AML | n.d. | AML | MPN/AML | AML | AML | AML | n.d. | n.d. | n.d. | n.d. | n.d. | n.d. | MPN | n.d. | T-ALL | AML |
| **Secondary Transplants** | **No. of mice** | 0 | 6 | 5 | 3 | 3 | 0 | 0 | 0 | 0 | 0 | 0 | 0 | 0 | 0 | 7 | 0 | 6 | 4 |
|  | **No. of mice dying from disease** | 0 | 6 | 0 | 3 | 3 | 0 | 0 | 0 | 0 | 0 | 0 | 0 | 0 | 0 | 7 | 0 | 6 | 4 |
|  | **Median time of survival (d)** | N/A | 35 (6) | N/A | 35 (3) | 65 (3) | N/A | N/A | N/A | N/A | N/A | N/A | N/A | N/A | N/A | 58 (7) | N/A | 21.5 (6) | 30 (4) |
|  |  |  |  |  |  |  |  |  |  |  |  |  |  |  |  |  |  |  |  |
|  |  |  |  |  |  |  |  |  |  |  |  |  |  |  |  |  |  |  |  |
| **Construct** | | **CTL** | **MN1** | **MN1 Δ1** | **MN1 Δ2** | **MN1 Δ4** | **MN1 Δ5** | **MN1 Δ6** | **MN1 Δ7** | **MN1 Δ1-2** | **MN1 Δ1-3** | **MN1 Δ1-4** | **MN1 Δ1-5** | **MN1 Δ1-6** | **MN1 Δ2-7** | **MN1 Δ3-7** | **MN1 Δ4-7** | **MN1 Δ5-7** | **MN1 Δ6-7** |
| **Secondary Transplants** | **Engraftment in BM at death (% GFP)** | N/A | 82.8 ± 15.4 (3) | 30.1 ± 12.6 (5) | n.d. | 62.9 ± 2.0 (3) | N/A | N/A | N/A | N/A | N/A | N/A | N/A | N/A | N/A | 87.4 ± 5.3 (3) | N/A | 67.5 ± 27.4 (3) | n.d. |
