# Supplementary material for: Cell Fate Decisions in Malignant Hematopoiesis: Leukemia Phenotype Is Determined by Distinct Functional Domains of the MN1 Oncogene
Source: PLoS One. 2014 Nov 17;9(11):e112671. doi: 10.1371/journal.pone.0112671 (PMC4234417; doi:10.1371/journal.pone.0112671)
Supplement: Table S4 — In vivo engraftment of cells transduced with MN1 deletion constructs. (DOC) [file pone.0112671.s013.doc]

**Supplementary Tables**

**Cell fate decisions in malignant hematopoiesis: Leukemia phenotype is determined by distinct functional domains of the MN1 oncogene**

Courteney K. Lai^1,2^, Yeonsook Moon^3^, Florian Kuchenbauer^4,5^, Daniel T. Starzcynowski^6^, Bob Argiropoulos^7^, Eric Yung^1^, Philip Beer^1^, Adrian Schwarzer^8^, Amit Sharma^8^, Gyeongsin Park^9^, Malina Leung^1^, Grace Lin^1^, Sarah Vollett^1^, Stephen Fung^1^, Connie J. Eaves^1,2^, Aly Karsan^10,11^, Andrew P. Weng^1,11^, R. Keith Humphries^1,2#^, Michael Heuser^12#^

**Table S4. *In vivo*** engraftment of cells transduced with MN1 deletion constructs.

| **Construct** | **No of Mice** | **Engraftment in Peripheral Blood (% GFP)** | | | | **Engraftment in RBCs (% GFP)** | | | **Engraftment in RBCs (% GFP) / WBC (% GFP)** | | |
| --- | --- | --- | --- | --- | --- | --- | --- | --- | --- | --- | --- |
|  |  | **Wk 4** | **Wk 8** | **Wk 12** | **Wk 16** | **Wk 4** | **Wk 12** | **Wk 16** | **Wk 4** | **Wk 12** | **Wk 16** |
| **CTL** | 2 | 7.66 | 3.01 ± 1.46 | 1.31 ± 1.06 | 1.88 ± 0.86 | 4.94 | 0.09 ± 0.01 | 0.08 ± 0.01 | 0.64 | 0.07 ± 0.17 | 0.04 ± 0.03 |
| **MN1** | 5 | 35.00 ± 10.19 | 12.61 ± 3.59 | n.d. | n.d. | 14.13 ± 3.63 | n.d. | n.d. | 0.40 ± 0.19 | n.d. | n.d. |
| **MN1Δ1** | 5 | 40.44 ± 6.49 | 23.64 ± 2.09 | 15.08 ± 4.23 | 13.28 ± 3.44 | 21.67 ± 8.80 | 81.90 ± 0.40 | 91.65 ± 0.45 | 0.54 ± 0.27 | 5.43 ± 0.84 | 6.90 ± 1.20 |
| **MN1Δ2** | 3 | 84.07 ± 1.36 | 86.45 ± 5.95 | n.d. | n.d. | 73.73 ± 2.91 | n.d. | n.d. | 0.88 ± 0.05 | n.d. | n.d. |
| **MN1Δ4** | 5 | 57.00 ± 7.50 | 66.04 ± 3.16 | 57.05 ± 6.05 | n.d. | 75.55 ± 2.55 | n.d. | n.d. | 1.33 | n.d. | n.d. |
| **MN1Δ5** | 3 | 40.43 ± 5.72 | 27.90 ± 2.89 | 62.43 ± 15.09 | 54.65 ± 20.45 | 32.80 ± 11.24 | 88.67 ± 3.51 | 91.60 ± 2.70 | 0.81 ± 0.20 | 1.42 ± 0.30 | 1.68 ± 0.79 |
| **MN1Δ6** | 9 | 18.42 ± 4.47 | 15.63 ± 6.31 | 19.95 ± 6.90 | 42.40 ± 13.19 | 47.29 ± 10.29 | 2.29 ± 1.63 | 3.11 ± 1.69 | 2.57 ± 0.48 | 0.11 ± 0.14 | 0.16 ± 0.64 |
| **MN1Δ7** | 5 | 78.30 ± 1.11 | 71.30 ± 14.64 | n.d. | n.d. | 14.60 ± 5.61 | n.d. | n.d. | 0.19 ± 0.07 | n.d. | n.d. |
| **MN1Δ1-2** | 4 | 11.69 ± 2.37 | 6.93 ± 1.64 | 3.47 ± 1.42 | 3.03 ± 1.24 | 10.42 ± 0.96 | 3.62 ± 3.18 | 3.70 ± 3.65 | 0.89 ± 0.20 | 1.04 ± 0.49 | 1.22 |
| **MN1Δ1-3** | 3 | n.d. | 2.10 ± 0.91 | 0.80 ± 0.18 | 0.19 | 2.47 ± 1.21 | n.d. | n.d. | n.d. | n.d. | n.d. |
| **MN1Δ1-4** | 5 | 1.95 ± 0.29 | 0.99 ± 0.18 | 0.40 ± 0.16 | 0.17 ± 0.2 | 0.15 ± 0.07 | n.d. | n.d. | 0.08 ± 0.04 | n.d. | n.d. |
| **MN1Δ1-5** | 5 | 8.05 ± 1.87 | 4.66 ± 1.00 | 2.38 ± 0.84 | 1.29 ± 0.53 | 0.91 ± 0.34 | n.d. | n.d. | 0.11 ± 0.20 | n.d. | n.d. |
| **MN1Δ1-6** | 3 | 15.23 ± 2.36 | 5.90 ± 1.08 | 3.50 ± 0.89 | 2.16 ± 0.74 | 5.41 ± 1.22 | n.d. | n.d. | 0.35 ± 0.03 | n.d. | n.d. |
| **MN1Δ2-7** | 2 | 4.47 ± 0.63 | 4.32 ± 0.49 | 2.47 ± 0.08 | 2.19 ± 1.55 | 2.97 ± 2.77 | n.d. | n.d. | 0.66 ± 0.73 | n.d. | n.d. |
| **MN1Δ3-7** | 3 | 69.20 ± 2.87 | 26.43 ± 7.44 | 33.88 ± 25.22 | 29.69 ± 24.60 | 1.98 ± 0.25 | n.d. | n.d. | 0.03 ± 0.00 | n.d. | n.d. |
| **MN1Δ4-7** | 4 | 26.37 ± 8.37 | 3.00 ± 2.27 | 7.34 ± 4.83 | 8.61 ± 2.85 | 10.67 ± 3.20 | 1.09 | 3.42 ± 3.09 | 0.40 ± 0.18 | 0.15 ± 0.02 | 0.40 |
| **MN1Δ5-7** | 8 | 11.31 ± 3.00 | 14.91 ± 4.63 | 13.11 ± 9.71 | 19.05 ± 9.91 | 18.17 ± 7.51 | 3.67 ± 3.58 | 0.00 | 1.61 ± 1.13 | 0.28 ± 0.14 | 0.00 |
| **MN1Δ6-7** | 5 | 39.28 ± 6.18 | 18.75 ± 5.95 | 6.17 ± 2.02 | 13.14 ± 5.57 | 17.56 ± 2.97 | 15.95 ± 0.45 | 0.28 ± 0.21 | 0.45 ± 0.12 | 2.59 ± 0.87 | 0.02 ± 0.01 |
