# Supplementary material for: Cell Fate Decisions in Malignant Hematopoiesis: Leukemia Phenotype Is Determined by Distinct Functional Domains of the MN1 Oncogene
Source: PLoS One. 2014 Nov 17;9(11):e112671. doi: 10.1371/journal.pone.0112671 (PMC4234417; doi:10.1371/journal.pone.0112671)
Supplement: Table S5 — Peripheral blood counts in mice receiving transplants of cells transduced with MN1 deletion constructs. (DOC) [file pone.0112671.s014.doc]

**Supplementary Tables**

**Cell fate decisions in malignant hematopoiesis: Leukemia phenotype is determined by distinct functional domains of the MN1 oncogene**

Courteney K. Lai^1,2^, Yeonsook Moon^3^, Florian Kuchenbauer^4,5^, Daniel T. Starzcynowski^6^, Bob Argiropoulos^7^, Eric Yung^1^, Philip Beer^1^, Adrian Schwarzer^8^, Amit Sharma^8^, Gyeongsin Park^9^, Malina Leung^1^, Grace Lin^1^, Sarah Vollett^1^, Stephen Fung^1^, Connie J. Eaves^1,2^, Aly Karsan^10,11^, Andrew P. Weng^1,11^, R. Keith Humphries^1,2#^, Michael Heuser^12#^

**Table S5**. Peripheral blood counts in mice receiving transplants of cells transduced with MN1 deletion constructs.

| **Construct** | **No of Mice** | **WBC (x10^3^/mm^3^)** | | | | | **RBC (x10^6^/mm^3^)** | | | | | **Hemoglobin (g/dl)** | | | | | **Platelets (x10^3^/mm^3^)** | | | | |
| --- | --- | --- | --- | --- | --- | --- | --- | --- | --- | --- | --- | --- | --- | --- | --- | --- | --- | --- | --- | --- | --- |
|  |  | **Wk 4** | **Wk 8** | **Wk 12** | **Wk 16** | **Wk 20** | **Wk 4** | **Wk 8** | **Wk 12** | **Wk 16** | **Wk 20** | **Wk 4** | **Wk 8** | **Wk 12** | **Wk 16** | **Wk 20** | **Wk 4** | **Wk 8** | **Wk 12** | **Wk 16** | **Wk 20** |
| **CTL** | 2 | 3.35 ± 0.55 | 4.80 ± 0.40 | 6.00 ± 0.60 | 8.15 ± 1.75 | n.d. | 6.06 ± 2.96 | 9.41 ± 0.10 | 8.97 ± 0.02 | 9.05 ± 0.77 | n.d. | 9.60 ± 4.70 | 13.15 ± 0.35 | 13.10 ± 0.10 | 13.55 ± 1.35 | n.d. | 406.00 ± 232.00 | 693.00 ± 117.00 | 743.00 ± 7.00 | 1086.00 ± 138.00 | n.d. |
| **MN1** | 5 | 6.34 ± 1.01 | n.d. | n.d. | n.d. | n.d. | 5.99 ± 1.00 | n.d. | n.d. | n.d. | n.d. | 8.94 ±1.50 | n.d. | n.d. | n.d. | n.d. | 148.00 ±26.13 | n.d. | n.d. | n.d. | n.d. |
| **MN1 Δ1** | 5 | 5.06 ± 0.65 | 9.62 ± 0.96 | 6.86 ± 1.34 | 7.08 ± 0.70 | 5.42 ± 0.53 | 8.05 ± 0.92 | 8.84 ± 0.81 | 9.07 ± 0.21 | 9.45 ± 0.42 | 6.24 ± 0.39 | 12.42 ± 1.33 | 12.28 ± 0.55 | 11.98 ± 0.39 | 12.72 ± 0.69 | 9.42 ± 0.56 | 411.20 ± 81.49 | 579.20 ± 67.90 | 792.80 ± 79.24 | 1007.80 ± 126.23 | 415.40 ± 161.95 |
| **MN1 Δ2** | 3 | 16.00 ± 0.61 | 33.47 ± 13.18 | n.d. | n.d. | n.d. | 5.85 ± 0.60 | 5.78 ± 0.94 | n.d. | n.d. | n.d. | 9.90 ± 0.97 | 11.47 ± 1.74 | n.d. | n.d. | n.d. | 186.00 ± 24.58 | 159.67 ± 54.19 | n.d. | n.d. | n.d. |
| **MN1 Δ4** | 2 | 2.50 ± 0.10 | 4.20 ± 0.60 | 19.05 ± 3.45 | n.d. | n.d. | 9.35 ± 0.14 | 4.28 ± 0.01 | 3.34 ± 1.20 | n.d. | n.d. | 13.05 ± 0.25 | 6.85 ± 0.25 | 5.65 ± 1.25 | n.d. | n.d. | 147.00 ± 15.00 | 128.50 ± 26.50 | 114.50 ± 79.50 | n.d. | n.d. |
| **MN1 Δ5** | 3 | 5.07 ± 0.94 | 5.23 ± 0.61 | 13.40 ± 9.46 | 12.73 ± 4.89 | n.d. | 6.51 ± 1.90 | 8.65 ± 1.09 | 6.22 ± 1.20 | 7.30 ± 1.27 | n.d. | 10.00 ± 2.67 | 11.80 ± 1.42 | 8.93 ± 1.30 | 10.20 ± 0.74 | n.d. | 273.00 ± 69.48 | 197.00 ± 37.99 | 102.00 ± 8.50 | 431.67 ± 343.32 | n.d. |
| **MN1 Δ6** | 9 | 4.79 ± 0.58 | 4.59 ± 0.56 | 3.88 ± 0.82 | 6.74 ± 0.52 | 0.82 | 8.60 ± 0.71 | 8.20 ± 0.79 | 6.44 ± 1.06 | 6.91 ± 1.45 | n.d. | 12.94 ± 1.05 | 11.42 ± 0.96 | 8.81 ± 1.71 | 10.74 ± 1.98 | n.d. | 353.78 ± 70.56 | 313.22 ± 63.98 | 322.89 ± 91.14 | 445.00 ± 171.72 | n.d. |
| **MN1 Δ7** | 5 | 4.08 ± 0.40 | 106.72 ± 58.00 | n.d. | n.d. | n.d. | 7.95 ± 0.28 | 6.63 ± 1.37 | n.d. | n.d. | n.d. | 12.74 ± 0.38 | 11.06 ± 2.03 | n.d. | n.d. | n.d. | 397.60 ± 98.13 | 349.80 ± 100.31 | n.d. | n.d. | n.d. |
| **MN1**  **Δ1-2** | 4 | 3.80 ± 0.38 | 5.68 ± 0.71 | 8.13 ± 0.50 | 8.73 ±0.74 | n.d. | 8.69 ± 0.90 | 10.23 ± 0.28 | 9.17 ± 0.35 | 9.40 ± 0.14 | n.d. | 13.10 ± 1.52 | 14.18 ± 0.27 | 13.33 ± 0.52 | 14.07 ± 0.35 | n.d. | 647.75 ± 131.82 | 627.50 ± 38.50 | 653.67 ± 11.86 | 877.33 ± 15.38 | n.d. |
| **MN1**  **Δ1-3** | 3 | 6.30 ± 2.42 | 7.07 ± 0.64 | 8.67 ± 0.90 | 13.40 | n.d. | 9.23 ± 0.04 | 10.53 ± 0.53 | 10.37 ± 0.79 | 10.98 | n.d. | 13.73 ± 0.12 | 15.67 ± 0.76 | 14.37 ± 0.99 | 15.60 | n.d. | 534.00 ± 59.56 | 540.67 ± 88.91 | 591.00 ± 57.49 | 888.00 | n.d. |
| **MN1**  **Δ1-4** | 5 | 2.50 ± 0.29 | 6.58 ± 0.78 | 3.78 ± 0.58 | 8.20 ± 1.39 | n.d. | 7.93 ± 0.80 | 9.87 ± 0.81 | 9.29 ± 0.26 | 9.17 ± 0.50 | n.d. | 11.98 ± 1.00 | 15.30 ± 1.46 | 13.05 ± 0.19 | 13.50 ± 0.39 | n.d. | 284.40 ± 58.38 | 454.60 ± 88.87 | 544.00 ± 141.55 | 597.40 ± 141.54 | n.d. |
| **MN1**  **Δ1-5** | 5 | 5.18 ± 1.05 | 7.24 ± 0.66 | 3.88 ± 0.85 | 7.56 ± 0.38 | n.d. | 8.46 ± 0.41 | 10.55 ± 0.23 | 9.63 ± 0.21 | 9.88 ± 0.41 | n.d. | 12.90 ± 0.56 | 15.58 ± 0.49 | 13.06 ± 0.13 | 14.08 ± 0.28 | n.d. | 545.20 ± 31.72 | 603.40 ± 34.92 | 646.60 ± 64.28 | 884.20 ± 64.02 | n.d. |
| **MN1**  **Δ1-6** | 3 | 3.50 ± 0.55 | 8.43 ± 2.06 | 9.10 ± 0.75 | 7.67 ± 1.99 | n.d. | 8.53 ± 0.19 | 9.29 ± 0.16 | 9.90 ± 0.09 | 9.33 ± 0.41 | n.d. | 12.80 ± 0.06 | 14.07 ± 0.12 | 13.87 ± 0.09 | 13.23 ± 0.58 | n.d. | 461.67 ± 75.50 | 770.33 ± 148.71 | 674.33 ± 42.01 | 789.67 ± 192.61 n.d. | n.d. |
| **MN1**  **Δ2-7** | 2 | 5.45 ± 3.65 | 6.70 ± 3.00 | 6.40 ± 1.70 | 3.45 ± 0.05 | n.d. | 9.06 ± 0.28 | 9.67 ± 0.27 | 10.69 ± 0.04 | 9.23 ± 0.40 | n.d. | 13.65 ± 0.75 | 14.90 ± 0.30 | 14.85 ± 0.15 | 13.40 ± 0.60 | n.d. | 286.00 ± 26.00 | 570.50 ± 94.50 | 578.00 ± 87.00 | 690.00 ± 47.00 | n.d. |
| **MN1**  **Δ3-7** | 3 | 7.07 ± 0.87 | 4.83 ± 0.90 | 9.07 ± 0.91 | 12.10 ± 3.20 | n.d. | 8.26 ± 0.45 | 7.50 ± 1.61 | 8.72 ± 0.44 | 9.04 ± 0.50 | n.d. | 13.07 ± 0.35 | 11.60 ± 2.35 | 12.27 ± 0.45 | 12.97 ± 0.44 | n.d. | 360.33 ± 34.23 | 690.00 ± 68.54 | 560.33 ± 119.56 | 740.67 ± 71.51 | n.d. |
| **MN1**  **Δ4-7** | 4 | 4.03 ± 0.66 | 3.80 ± 0.31 | 4.40 ± 0.71 | 6.90 ± 0.86 | n.d. | 9.57 ± 0.43 | 10.93 ± 0.64 | 8.97 ± 0.14 | 9.96 ± 0.26 | n.d. | 15.18 ± 0.67 | 15.58 ± 1.23 | 13.50 ± 0.21 | 15.15 ± 0.41 | n.d. | 731.75 ± 147.96 | 486.00 ± 37.25 | 582.75 ± 50.43 | 996.25 ± 69.13 | n.d. |
| **MN1**  **Δ5-7** | 8 | 4.29 ± 0.59 | 5.83 ± 0.78 | 5.14 ± 0.67 | 6.63 ± 0.96 | 3.67 ± 0.27 | 9.37 ± 0.23 | 10.17 ± 0.84 | 8.94 ± 0.49 | 9.21 ± 0.72 | 5.64 ± 0.90 | 14.04 ± 0.40 | 15.43 ± 1.20 | 12.32 ± 0.87 | 13.18 ± 1.18 | 9.20 ± 1.19 | 362.38 ± 65.08 | 514.83 ± 119.93 | 560.00 ± 56.52 | 845.25 ± 117.41 | 174.33 ± 41.07 |
| **MN1**  **Δ6-7** | 5 | 5.24 ± 0.81 | 4.80 ± 0.40 | 3.55 ± 1.25 | 4.60 ± 2.40 | n.d. | 7.37 ± 1.27 | 10.97 ± 0.55 | 6.84 ± 1.52 | 4.69 ± 3.37 | n.d. | 11.02 ± 1.81 | 15.50 ± 0.70 | 10.25 ± 1.85 | 7.65 ± 5.05 | n.d. | 269.00 ± 103.45 | 420.00 ± 181.00 | 465.65 ± 464.35 | 1098.00 ± 1071.00 | n.d. |
