# Supplementary material for: Cell Fate Decisions in Malignant Hematopoiesis: Leukemia Phenotype Is Determined by Distinct Functional Domains of the MN1 Oncogene
Source: PLoS One. 2014 Nov 17;9(11):e112671. doi: 10.1371/journal.pone.0112671 (PMC4234417; doi:10.1371/journal.pone.0112671)
Supplement: Table S6 — Immunophenotype of GFP-positive cells in peripheral blood of mice receiving transplants of cells transduced with MN1 deletion constructs. (DOC) [file pone.0112671.s015.doc]

**Supplementary Tables**

**Cell fate decisions in malignant hematopoiesis: Leukemia phenotype is determined by distinct functional domains of the MN1 oncogene**

Courteney K. Lai^1,2^, Yeonsook Moon^3^, Florian Kuchenbauer^4,5^, Daniel T. Starzcynowski^6^, Bob Argiropoulos^7^, Eric Yung^1^, Philip Beer^1^, Adrian Schwarzer^8^, Amit Sharma^8^, Gyeongsin Park^9^, Malina Leung^1^, Grace Lin^1^, Sarah Vollett^1^, Stephen Fung^1^, Connie J. Eaves^1,2^, Aly Karsan^10,11^, Andrew P. Weng^1,11^, R. Keith Humphries^1,2#^, Michael Heuser^12#^

**Table S6. Immuno**phenotype of GFP-positive cells in peripheral blood of mice receiving transplants of cells transduced with MN1 deletion constructs.

| **Construct** | | | | | | | | | | **CTL** | | | | **MN1** | | | | **MN1 Δ1** | | **MN1 Δ2** | | **MN1 Δ4** | | | **MN1 Δ5** | | | **MN1 Δ6** | | | **MN1 Δ7** | | **MN1 Δ1-2** | | | **MN1 Δ1-3** | | **MN1 Δ1-4** | | | **MN1 Δ1-5** | | **MN1 Δ1-6** | | | **MN1 Δ2-7** | | | **MN1 Δ3-7** | | | **MN1 Δ4-7** | | | **MN1 Δ5-7** | | | **MN1 Δ6-7** | |  |
| --- | --- | --- | --- | --- | --- | --- | --- | --- | --- | --- | --- | --- | --- | --- | --- | --- | --- | --- | --- | --- | --- | --- | --- | --- | --- | --- | --- | --- | --- | --- | --- | --- | --- | --- | --- | --- | --- | --- | --- | --- | --- | --- | --- | --- | --- | --- | --- | --- | --- | --- | --- | --- | --- | --- | --- | --- | --- | --- | --- | --- |
| **No of Mice** | | | | | | | | | | **2** | | | | **6** | | | | **2** | | **3** | | **2** | | | **3** | | | **9** | | | **5** | | **4** | | | **3** | | **5** | | | **5** | | **3** | | | **2** | | | **3** | | | **4** | | | **8** | | | **5** | |  |
| **% Gr1^+^** | | | **Week** | | | **4** | | | | 0.00 | | | | 11.78 ± 0.99 | | | | 17.49 ± 5.42 | | 64.63 ± 2.98 | | 11.37 ± 2.83 | | | 33.30 ± 4.76 | | | 32.24 ± 9.73 | | | 47.84 ± 11.78 | | 10.67 ± 3.25 | | | 16.07 ± 10.46 | | 0.72 | | | 7.45 ± 3.18 | | 3.32 ± 1.57 | | | n.d. | | | 16.93 ± 3.90 | | | 13.93 ± 7.22 | | | 10.88 ± 6.84 | | | 20.12 ± 11.81 | |  |
|  |  |  |  |  |  | **8** | | | | 0.54 ± 0.54 | | | | 1.99 ± 0.99 | | | | 1.94 ± 0.73 | | 27.95 ± 0.15 | | 20.85 ± 2.25 | | | 0.74 ± 0.18 | | | 8.60 ± 3.28 | | | 41.88 ± 10.73 | | 12.58 ± 7.27 | | | 2.78 ± 2.78 | | n.d. | | | 4.07 ± 0.86 | | 1.12 ± 0.37 | | | 2.84 ± 0.39 | | | 11.15 ± 8.08 | | | 6.68 ± 6.68 | | | n.d. | | | 15.66 ± 14.25 | |  |
|  |  |  |  |  |  | **12** | | | | 16.65 ± 16.65 | | | | n.d. | | | | 18.62 ± 11.59 | | n.d. | | 0.45 ± 0.04 | | | 23.35 ± 8.89 | | | 17.35 ± 10.15 | | | n.d. | | n.d. | | | 0.00 ± 0.00 | | 0.00 | | | 0.29 ± 0.29 | | 0.53 ± 0.22 | | | 2.44 ± 2.44 | | | 0.59 ± 0.33 | | | 18.50 ± 16.27 | | | 0.14 ± 0.14 | | | 0.00 ± 0.00 | |  |
|  |  |  |  |  |  | **16** | | | | n.d. | | | | n.d. | | | | 13.61 ± 9.62 | | n.d. | | n.d. | | | 5.89 ± 5.42 | | | 0.11 | | | n.d. | | 3.86 ± 1.15 | | | n.d. | | n.d. | | | n.d. | | n.d. | | | n.d. | | | 33.54 ± 27.66 | | | 50.90 | | | 7.93 ± 3.08 | | | 21.90 ± 11.40 | |  |
| **% CD11b^+^** | | | **Week** | | | **4** | | | | 6.82 | | | | 28.80 ± 4.53 | | | | 25.04 ± 8.40 | | 78.50 ± 3.50 | | 23.65 ± 4.15 | | | 49.80 ± 6.11 | | | 35.74 ± 9.65 | | | 62.92 ± 12.75 | | 19.33 ± 4.89 | | | 25.00 ± 14.43 | | n.d. | | | 8.60 ± 1.64 | | 8.47 ± 1.15 | | | n.d. | | | 27.80 ± 3.27 | | | 20.35 ± 8.86 | | | 12.25 ± 7.37 | | | 29.54 ± 13.68 | |  |
|  |  |  |  |  |  | **8** | | | | 4.17 ± 4.17 | | | | 14.45 ± 4.25 | | | | 3.25 ± 0.51 | | 83.85 ± 7.25 | | 28.50 ± 2.30 | | | 9.04 ± 3.13 | | | 10.23 ± 3.04 | | | 83.18 ± 5.28 | | 20.42 ± 10.85 | | | 0.00 ± 0.00 | | n.d. | | | 2.97 ± 1.31 | | 1.79 ± 0.46 | | | 5.28 ± 2.05 | | | 10.39 ± 7.37 | | | 11.43 ± 3.38 | | | n.d. | | | 20.47 ± 17.43 | |  |
|  |  |  |  |  |  | **12** | | | | 19.78 ± 13.53 | | | | n.d. | | | | 22.63 ± 13.18 | | n.d. | | 19.35 ± 1.45 | | | 32.07 ± 11.82 | | | 24.72 ± 10.80 | | | n.d. | | n.d. | | | 1.45 ± 1.45 | | 33.30 | | | 0.98 ± 0.57 | | 3.00 ± 0.66 | | | 1.45 ± 1.45 | | | 18.67 ± 16.66 | | | 33.17 ± 16.00 | | | 2.70 ± 1.48 | | | 18.13 ± 12.77 | |  |
|  |  |  |  |  |  | **16** | | | | 0.00 | | | | n.d. | | | | 39.57 ± 20.60 | | n.d. | | n.d. | | | 15.93 ± 8.28 | | | 25.80 ± 8.14 | | | n.d. | | 10.13 ± 6.31 | | | n.d. | | n.d. | | | n.d. | | n.d. | | | n.d. | | | 48.29 ± 42.41 | | | 54.70 | | | 15.39 ± 2.78 | | | 23.25 ± 10.05 | |  |
|  | | |  | | |  | | | |  | | | |  | | | |  | |  | |  | | |  | | |  | | |  | |  | | |  | |  | | |  | |  | | |  | | |  | | |  | | |  | | |  | | |
|  | | |  | | |  | | | |  | | | |  | | | |  | |  | |  | | |  | | |  | | |  | |  | | |  | |  | | |  | |  | | |  | | |  | | |  | | |  | | |  | | |
|  | | |  | | |  | | | |  | | | |  | | | |  | |  | |  | | |  | | |  | | |  | |  | | |  | |  | | |  | |  | | |  | | |  | | |  | | |  | | |  | | |
|  | | |  | | |  | | | |  | | | |  | | | |  | |  | |  | | |  | | |  | | |  | |  | | |  | |  | | |  | |  | | |  | | |  | | |  | | |  | | |  | | |
|  | | |  | | |  | | | |  | | | |  | | | |  | |  | |  | | |  | | |  | | |  | |  | | |  | |  | | |  | |  | | |  | | |  | | |  | | |  | | |  | | |
| **Construct** | | | | | | | | **CTL** | | | | **MN1** | | | | **MN1 Δ1** | | | **MN1 Δ2** | | **MN1 Δ4** | | | **MN1 Δ5** | | | **MN1 Δ6** | | | **MN1 Δ7** | | **MN1 Δ1-2** | | | **MN1 Δ1-3** | | **MN1 Δ1-4** | | **MN1 Δ1-5** | | | **MN1 Δ1-6** | | **MN1 Δ2-7** | | | **MN1 Δ3-7** | | | **MN1 Δ4-7** | | | **MN1 Δ5-7** | | | **MN1 Δ6-7** | | |  | |
| **% Gr1^+^/ CD11b^+^** | **Week** | | | **4** | | | 0.00 | | | | 12.28 ± 0.97 | | | | 15.18 ± 6.00 | | | | 65.33 ± 2.90 | | 11.37 ± 2.83 | | 32.80 ± 4.80 | | | 20.05 ± 8.73 | | | 48.44 ± 12.00 | | | 10.30 ± 3.17 | | 11.90 ± 11.90 | | | n.d. | | 6.08 ± 1.83 | | | 3.13 ± 2.21 | | n.d. | | | 20.25 ± 3.55 | | | 11.53 ± 7.88 | | | 10.68 ± .77 | | | 20.16 ± 11.92 | | |  | |
|  |  |  |  | **8** | | | 4.44 ± 3.90 | | | | 1.68 ± 0.17 | | | | 9.18 ± 5.02 | | | | 28.80 ± 0.20 | | 20.40 ± 2.70 | | 33.97 ± 17.19 | | | 27.04 ± 9.78 | | | 45.53 ± 10.55 | | | 12.93 ± 7.60 | | 0.00 ± 0.00 | | | n.d. | | 0.78 ± 0.78 | | | 0.61 ± 0.29 | | 1.62 ± 1.62 | | | 9.30 ± 7.35 | | | 25.81 ± 15.77 | | | n.d. | | | 15.44 ± 14.27 | | |  | |
|  |  |  |  | **12** | | | 16.65 ± 16.65 | | | | n.d. | | | | 28.59 ± 16.46 | | | | n.d. | | 23.95 ± 2.85 | | 24.12 ± 9.00 | | | 20.82 ± 9.95 | | | n.d. | | | n.d. | | 0.00 ± 0.00 | | | 0.00 | | 0.17 ± 0.17 | | | 0.14 ± 0.11 | | 0.00 ± 0.00 | | | 38.77 ± 23.71 | | | 18.80 ± 16.57 | | | 39.04 ± 13.89 | | | 0.00 ± 0.00 | | |  | |
|  |  |  |  | **16** | | | 0.00 | | | | n.d. | | | | 18.30 ± 11.29 | | | | n.d. | | n.d. | | 4.06 ± 3.21 | | | 11.48 ± 3.98 | | | n.d. | | | 2.01 ± 1.27 | | n.d. | | | n.d. | | n.d. | | | n.d. | | n.d. | | | 37.64 ± 31.76 | | | 27.57 ± 14.85 | | | 6.35 ± 6.35 | | | 21.90 ± 11.40 | | |  | |
| **% cKit.** | **Week** | | | **4** | | | 0.00 | | | | 20.54 ± 7.76 | | | | 14.84 ± 14.57 | | | | 7.00 ± 1.78 | | 0.53 ± 0.53 | | 2.69 ± 0.81 | | | 7.27 ± 3.77 | | | 4.64 ± 1.89 | | | 2.12 ± 0.28 | | 2.08 ± 2.08 | | | n.d. | | 0.00 ± 0.00 | | | 0.35 ± 0.31 | | n.d. | | | 0.99 ± 0.33 | | | 13.90 ± 10.29 | | | 0.28 ± 0.17 | | | 46.68 ± 17.69 | | |  | |
|  |  |  |  | **8** | | | 0.24 ± 0.24 | | | | 71.60 ± 8.80 | | | | 0.42 ± 0.16 | | | | 1.30 | | 2.18 ± 2.18 | | 3.93 ± 1.96 | | | 19.83 ± 7.33 | | | 1.45 ± 0.60 | | | 0.10 ± 0.10 | | 0.00 | | | n.d. | | 0.80 ± 0.80 | | | 0.00 ± 0.00 | | 1.07 ± 1.07 | | | 0.30 ± 0.25 | | | 0.00 ± 0.00 | | | n.d. | | | 2.71 ± 2.47 | | |  | |
|  |  |  |  | **12** | | | 0.00 ± 0.00 | | | | n.d. | | | | 1.31 ± 0.61 | | | | n.d. | | 28.95 ± 17.85 | | 36.90 ± 13.84 | | | 29.52 ± 9.64 | | | n.d. | | | n.d. | | 0.00 ± 0.00 | | | 0.00 | | 0.46 ± 0.37 | | | 0.27 ± 0.21 | | 0.00 ± 0.00 | | | 0.59± 0.34 | | | 27.41 ± 26.50 | | | 0.18 ± 0.09 | | | 14.86 ± 9.14 | | |  | |
|  |  |  |  | **16** | | | 0.00 | | | | n.d. | | | | 6.50 ± 5.08 | | | | n.d. | | n.d. | | 57.20 ± 17.70 | | | 77.60 | | | n.d. | | | 3.34 ± 2.28 | | n.d. | | | n.d. | | n.d. | | | n.d. | | n.d. | | | 0.00 ± 0.00 | | | 2.78 ± 2.00 | | | 2.14 ± 1.08 | | | 43.96 ± 34.64 | | |  | |
| **% sca1^+^** | **Week** | | | **4** | | | 0.00 | | | | 40.06 ± 5.00 | | | | 58.38 ± 15.00 | | | | 5.61 ± 3.25 | | 34.40 ± 6.00 | | 53.63 ± 2.39 | | | 59.59 ± 9.27 | | | 24.28 ± 5.72 | | | 56.90 ± 4.26 | | 78.13 ± 9.68 | | | n.d. | | 65.70 ± 17.78 | | | 72.40 ± 5.51 | | n.d. | | | 56.67 ± 8.75 | | | 51.74 ± 25.41 | | | 55.18 ± 9.59 | | | 32.22 ± 9.32 | | |  | |
|  |  |  |  | **8** | | | 0.24 ± 0.24 | | | | 7.06 ± 2.79 | | | | 69.36 ± 9.59 | | | | 7.04 | | 34.50 ± 9.90 | | 42.47 ± 21.39 | | | 31.33 ± 8.59 | | | 3.77 ± 1.57 | | | 44.65 ± 4.26 | | 66.70 | | | n.d. | | 86.70 ± 4.20 | | | 85.20 ± 1.21 | | 89.45 ± 4.15 | | | 68.83 ± 12.40 | | | 51.17 ± 15.60 | | | n.d. | | | 64.15 ± 16.15 | | |  | |
|  |  |  |  | **12** | | | 0.00 ± 0.00 | | | | n.d. | | | | 44.90 ± 14.69 | | | | n.d. | | 8.64 ± 4.67 | | 33.40 ± 1.19 | | | 29.17 ± 11.81 | | | n.d. | | | n.d. | | 73.40 ± 11.17 | | | 60.00 | | 78.24 ± 5.69 | | | 55.97 ± 21.69 | | 76.60 ± 2.70 | | | 33.69 ± 22.53 | | | 28.73 ± 15.53 | | | 25.37 ± 6.21 | | | 35.20 ± 2.80 | | |  | |
|  |  |  |  | **16** | | | 88.90 | | | | n.d. | | | | 45.93 ± 13.99 | | | | n.d. | | n.d. | | 43.10 ± 12.70 | | | 53.90 ± 10.57 | | | n.d. | | | 72.87 ± 8.40 | | n.d. | | | n.d. | | n.d. | | | n.d. | | n.d. | | | 35.27 ± 30.63 | | | 55.63 ± 15.68 | | | 54.85 ± 8.65 | | | 54.70 ± 19.00 | | |  | |
| **% cKit +/ sca1^+^** | **Week** | | | **4** | | | 0.00 | | | | 8.10 ± 3.72 | | | | 0.67 ± 0.29 | | | | 0.48 ± 0.13 | | 0.53 ± 0.53 | | 2.16 ± 0.13 | | | 4.47 ± 3.12 | | | 2.20 ± 1.93 | | | 0.28 ± 0.07 | | 0.00 ± 0.00 | | | n.d. | | 0.00 ± 0.00 | | | 0.35 ± 0.31 | | n.d. | | | 0.78 ± 0.55 | | | 1.56 ± 1.17 | | | 0.15 ± 0.09 | | | 14.62 ± 7.29 | | |  | |
|  |  |  |  | **8** | | | 0.24 ± 0.24 | | | | 3.78 ± 1.30 | | | | 0.39 ± 0.26 | | | | 0.03 | | 0.00 ± 0.00 | | 5.52 ± 3.36 | | | 3.37 ± 1.50 | | | 0.20 ± 0.10 | | | 0.00 ± 0.00 | | 0.00 | | | n.d. | | 0.80 ± 0.80 | | | 0.16 ± 0.13 | | 0.00 ± 0.00 | | | 0.59 ± 0.59 | | | 0.00 ± 0.00 | | | n.d. | | | 0.90 ± 0.90 | | |  | |
|  |  |  |  | **12** | | | 0.00 ± 0.00 | | | | n.d. | | | | 0.22 ± 0.15 | | | | n.d. | | 2.37 ± 1.45 | | 15.80 ± 5.58 | | | 4.99 ± 1.43 | | | n.d. | | | n.d. | | 0.00 ± 0.00 | | | 0.00 | | 0.21 ± 0.21 | | | 0.00 ± 0.00 | | 0.73 ± 0.73 | | | 0.08 ± 0.06 | | | 0.06 ± 0.06 | | | 0.05 ± 0.05 | | | 1.09 ± 1.09 | | |  | |
|  |  |  |  | **16** | | | 0.00 | | | | n.d. | | | | 0.24 ± 0.24 | | | | n.d. | | n.d. | | 16.55 ± 2.55 | | | 13.21 ± 8.48 | | | n.d. | | | 0.00 ± 0.00 | | 0.00 ± 0.00 | | | n.d. | | n.d. | | | n.d. | | n.d. | | | 0.00 ± 0.00 | | | 0.56 ± 0.56 | | | 1.36 ± 0.46 | | | 12.82 ± 8.58 | | |  | |
|  | |  | | |  | | | |  | | | |  | | | |  | |  | |  | | |  | |  | | |  | | |  | |  | | |  | | |  | |  | | |  | | |  | | |  | | |  | | |  | | | |
|  | |  | | |  | | | |  | | | |  | | | |  | |  | |  | | |  | |  | | |  | | |  | |  | | |  | | |  | |  | | |  | | |  | | |  | | |  | | |  | | | |
|  | |  | | |  | | | |  | | | |  | | | |  | |  | |  | | |  | |  | | |  | | |  | |  | | |  | | |  | |  | | |  | | |  | | |  | | |  | | |  | | | |
|  | |  | | |  | | | |  | | | |  | | | |  | |  | |  | | |  | |  | | |  | | |  | |  | | |  | | |  | |  | | |  | | |  | | |  | | |  | | |  | | | |

| **Construct** | | | **CTL** | **MN1** | **MN1 Δ1** | **MN1 Δ2** | **MN1 Δ4** | **MN1 Δ5** | **MN1 Δ6** | **MN1 Δ7** | **MN1 Δ1-2** | **MN1 Δ1-3** | **MN1 Δ1-4** | **MN1 Δ1-5** | **MN1 Δ1-6** | **MN1 Δ2-7** | **MN1 Δ3-7** | **MN1 Δ4-7** | **MN1 Δ5-7** | **MN1 Δ6-7** |
| --- | --- | --- | --- | --- | --- | --- | --- | --- | --- | --- | --- | --- | --- | --- | --- | --- | --- | --- | --- | --- |
| **% CD4^+^** | **Week** | **4** | 5.56 | 1.52 ± 0.49 | 11.91 ± 2.50 | 4.75 ± 0.19 | 3.21 ± 1.06 | 4.32 ± 1.86 | 20.90 ± 6.56 | 2.76 ± 1.02 | 9.48 ± 3.51 | 9.44 ± 3.78 | n.d. | 18.56 ± 8.93 | 5.80 ± 2.61 | n.d. | 20.47 ± 19.01 | 3.27 ± 2.26 | 15.62 ± 9.00 | 0.48 ± 0.36 |
|  |  | **8** | 24.67 ± 17.53 | 0.62 ± 0.20 | 23.56 ± 7.56 | 1.76 ± 0.98 | 6.37 ± 0.30 | 5.88 ± 1.56 | 6.22 ± 2.82 | 0.26 ± 0.09 | 29.30 ± 9.28 | 15.40 | n.d. | 22.07 ± 17.53 | 12.05 ± 1.24 | 13.85 ± 11.15 | 12.57 ± 3.49 | 24.54 ± 8.13 | n.d. | 2.92 ± 0.93 |
|  |  | **12** | 22.75 ± 22.75 | n.d. | 35.18 ± 13.01 | n.d. | 7.59 ± 5.72 | 6.43 ± 2.98 | 11.12 ± 6.18 | n.d. | n.d. | 26.80 ± 26.80 | 41.70 | 24.99 ± 7.75 | 5.96 ± 1.82 | 18.09 ± 11.42 | 14.60 ± 12.20 | 27.36 ± 10.62 | 7.10 ± 2.68 | 3.39 |
|  |  | **16** | 41.70 | n.d. | 21.41 ± 11.52 | n.d. | n.d. | 2.74 ± 0.49 | 10.48 ± 2.69 | n.d. | 43.30 ± 12.86 | n.d. | n.d. | n.d. | n.d. | n.d. | 5.63 ± 4.87 | 19.37 ± 8.26 | 22.68 ± 1.97 | 3.95 ± 3.95 |
| **% CD8^+^** | **Week** | **4** | 2.78 | 0.37 ± 0.18 | 1.79 ± 1.11 | 4.75 ± 0.19 | 0.22 ± 0.22 | 1.97 ± 1.05 | 1.41 ± 0.69 | 1.87 ± 0.32 | 6.35 ± 2.21 | 0.00 ± 0.00 | n.d. | 4.43 ± 3.80 | 2.39 ± 0.85 | n.d. | 0.75 ± 0.12 | 0.58 ± 0.58 | 2.89 ± 1.29 | 0.15 ± 0.10 |
|  |  | **8** | 7.10 ± 7.10 | 0.12 ± 0.12 | 8.82 ± 1.99 | 1.76 ± 0.98 | 2.98 ± 1.46 | 5.16 ± 0.88 | 2.08 ± 1.03 | 0.53 ± 0.22 | 18.93 ± 6.16 | 15.40 | n.d. | 7.46 ± 6.31 | 5.60 ± 1.47 | 7.80 ± 7.80 | 5.75 ± 1.25 | 11.04 ± 6.11 | n.d. | 1.20 ± 0.25 |
|  |  | **12** | 4.55 ± 4.55 | n.d. | 10.88 ± 4.25 | n.d. | 2.19 ± 1.75 | 3.08 ± 1.26 | 3.62 ± 1.34 | n.d. | n.d. | 13.30 ± 13.30 | 16.70 | 12.56 ± 3.43 | 6.30 ± 1.10 | 9.27 ± 5.94 | 9.06 ± 6.53 | 10.18 ± 3.01 | 6.60 ± 2.61 | 1.69 |
|  |  | **16** | 16.70 | n.d. | 3.89 ± 2.82 | n.d. | n.d. | 2.38 ± 0.85 | 8.69 ± 5.76 | n.d. | 16.88 ± 5.88 | n.d. | n.d. | n.d. | n.d. | n.d. | 5.01 ± 4.51 | 11.05 ± 3.26 | 21.83 ± 5.89 | 1.32 ± 1.32 |
| **% CD4^+^/ CD8^+^** | **Week** | **4** | 0.00 | 0.08 ± 0.04 | 0.36 ± 0.32 | 1.10 ± 0.29 | 0.00 ± 0.00 | 0.11 ± 0.04 | 0.00 ± 0.00 | 0.00 ± 0.00 | 0.30 ±0.16 | 0.00 ± 0.00 | n.d. | 0.00 ± 0.00 | 0.00 ± 0.00 | n.d. | 0.14 ± 0.14 | 0.00 ± 0.00 | 0.00 ± 0.00 | 0.00 ± 0.00 |
|  |  | **8** | 0.24 ± 0.24 | 0.00 ± 0.00 | 0.22 ± 0.08 | 1.04 ± 0.70 | 0.00 ± 0.00 | 0.23 ± 0.17 | 0.03 ± 0.02 | 0.02 ± 0.01 | 0.00 ± 0.00 | 0.00 | n.d. | 1.11 ± 1.11 | 1.37 ± 0.70 | 0.00 ± 0.00 | 0.08 ± 0.08 | 2.50 ± 2.50 | n.d. | 0.00 ± 0.00 |
|  |  | **12** | 0.00 ± 0.00 | n.d. | 0.18 ± 0.18 | n.d. | 0.09 ± 0.09 | 0.07 ± 0.06 | 2.60 ± 2.57 | n.d. | n.d. | 0.00 ± 0.00 | 0.00 | 0.20 ± 0.20 | 0.00 ± 0.00 | 0.48 ± 0.48 | 0.02 ± 0.02 | 0.65 ± 0.65 | 0.11 ± 0.07 | 0.00 |
|  |  | **16** | 0.00 | n.d. | 0.00 ± 0.00 | n.d. | n.d. | 0.00 ± 0.00 | 0.27 ± 0.27 | n.d. | 3.90 ± 3.90 | n.d. | n.d. | n.d. | n.d. | n.d. | 0.00 ± 0.00 | 0.00 ± 0.00 | 0.00 ± 0.00 | 0.44 ± 0.44 |
