# Supplementary material for: Cell Fate Decisions in Malignant Hematopoiesis: Leukemia Phenotype Is Determined by Distinct Functional Domains of the MN1 Oncogene
Source: PLoS One. 2014 Nov 17;9(11):e112671. doi: 10.1371/journal.pone.0112671 (PMC4234417; doi:10.1371/journal.pone.0112671)
Supplement: Table S7 — Gene ontology gene sets enriched in MN1Δ7 cells compared to MN1 cells. (DOC) [file pone.0112671.s016.doc]

**Supplementary Tables**

**Cell fate decisions in malignant hematopoiesis: Leukemia phenotype is determined by distinct functional domains of the MN1 oncogene**

Courteney K. Lai^1,2^, Yeonsook Moon^3^, Florian Kuchenbauer^4,5^, Daniel T. Starzcynowski^6^, Bob Argiropoulos^7^, Eric Yung^1^, Philip Beer^1^, Adrian Schwarzer^8^, Amit Sharma^8^, Gyeongsin Park^9^, Malina Leung^1^, Grace Lin^1^, Sarah Vollett^1^, Stephen Fung^1^, Connie J. Eaves^1,2^, Aly Karsan^10,11^, Andrew P. Weng^1,11^, R. Keith Humphries^1,2#^, Michael Heuser^12#^

**Table S7. Gene ontology gene sets enriched in MN1Δ**7 cells compared to MN1 cells.

| **Rank** | **Gene Sets** | **Normalised Enrichment Score (NES)** | **P** | **Category** |
| --- | --- | --- | --- | --- |
| 1 | OXYGEN_AND_REACTIVE_OXYGEN_SPECIES_METABOLIC_PROCESS | 1.45 | 0.0 | metabolism |
| 2 | REGULATION_OF_NEUROTRANSMITTER_LEVELS | 1.44 | 0.0 | signal transduction |
| 3 | CHEMOKINE_ACTIVITY | 1.40 | 0.0 | immune response / regulation |
| 4 | BIOGENIC_AMINE_METABOLIC_PROCESS | 1.36 | 0.0 | metabolism |
| 5 | G_PROTEIN_COUPLED_RECEPTOR_BINDING | 1.35 | 0.0 | signal transduction |
| 6 | CYTOKINE_ACTIVITY | 1.31 | 0.0 | immune response / regulation |
| 7 | VOLTAGE_GATED_CATION_CHANNEL_ACTIVITY | 1.31 | 0.0 | signal transduction |
| 8 | VOLTAGE_GATED_CHANNEL_ACTIVITY | 1.31 | 0.0 | signal transduction |
| 9 | VOLTAGE_GATED_POTASSIUM_CHANNEL_ACTIVITY | 1.30 | 0.0 | signal transduction |
| 10 | LIGAND_DEPENDENT_NUCLEAR_RECEPTOR_ACTIVITY | 1.30 | 0.0 | signal transduction |
| 11 | REGULATION_OF_BLOOD_PRESSURE | 1.30 | 0.0 | Other |
| 12 | AMINO_ACID_DERIVATIVE_METABOLIC_PROCESS | 1.30 | 0.0 | metabolism |
| 13 | EXOCYTOSIS | 1.29 | 0.0 | cell structure |
| 14 | CHEMOKINE_RECEPTOR_BINDING | 1.29 | 0.0 | immune response / regulation |
| **Rank** | **Gene Sets** | **Normalised Enrichment Score (NES)** | **P** | **Category** |
| 15 | HEMATOPOIETIN_INTERFERON_CLASSD200_DOMAIN_CYTOKINE_RECEPTOR_BINDING | 1.24 | 0.0 | immune response / regulation |
| 16 | HORMONE_METABOLIC_PROCESS | 1.23 | 0.2073922 | metabolism |
| 17 | TRANSCRIPTION_COFACTOR_ACTIVITY | 1.22 | 0.0 | transcription/translation |
| 18 | AXON_GUIDANCE | 1.22 | 0.0 | cell structure |
| 19 | GROWTH_FACTOR_ACTIVITY | 1.21 | 0.09128631 | immune response / regulation |
| 20 | MESODERM_DEVELOPMENT | 1.20 | 0.0 | cell differentiation |
| 21 | REGULATION_OF_DNA_METABOLIC_PROCESS | 1.20 | 0.26868686 | metabolism |
| 22 | REGULATION_OF_IMMUNE_RESPONSE | 1.20 | 0.09034908 | immune response / regulation |
| 23 | TRANSLATION | 1.19 | 0.09128631 | transcription/translation |
| 24 | TRANSCRIPTION_COREPRESSOR_ACTIVITY | 1.19 | 0.0 | transcription/translation |
| 25 | ANTIOXIDANT_ACTIVITY | 1.19 | 0.19507186 | metabolism |
| 26 | SODIUM_ION_TRANSPORT | 1.19 | 0.0 | signal transduction |
| 27 | NEGATIVE_REGULATION_OF_BIOSYNTHETIC_PROCESS | 1.19 | 0.0 | metabolism |
| 28 | REGULATION_OF_CELL_DIFFERENTIATION | 1.18 | 0.0 | cell differentiation |
| 29 | NEGATIVE_REGULATION_OF_CELLULAR_BIOSYNTHETIC_PROCESS | 1.18 | 0.0 | metabolism |
| 30 | RESPONSE_TO_OXIDATIVE_STRESS | 1.18 | 0.178 | metabolism |
| 31 | NUCLEAR_BODY | 1.18 | 0.0 | Other |
| 32 | NEGATIVE_REGULATION_OF_CELLULAR_PROTEIN_METABOLIC_PROCESS | 1.18 | 0.0 | metabolism |
| 33 | ACTIN_FILAMENT_ORGANIZATION | 1.18 | 0.0 | cell structure |
| 34 | ESTABLISHMENT_AND_OR_MAINTENANCE_OF_CELL_POLARITY | 1.18 | 0.0 | cell structure |
| 35 | TRANSCRIPTION_FACTOR_BINDING | 1.18 | 0.0 | transcription/translation |
| 36 | VIRAL_REPRODUCTIVE_PROCESS | 1.18 | 0.0 | immune response / regulation |
| 37 | SPECIFIC_RNA_POLYMERASE_II_TRANSCRIPTION_FACTOR_ACTIVITY | 1.18 | 0.0 | transcription/translation |
| 38 | NEGATIVE_REGULATION_OF_PROTEIN_METABOLIC_PROCESS | 1.17 | 0.0 | metabolism |
| 39 | GLYCOLIPID_METABOLIC_PROCESS | 1.17 | 0.0 | metabolism |
| 40 | CHROMOSOME | 1.17 | 0.0 | cell structure |
| **Rank** | **Gene Sets** | **Normalised Enrichment Score (NES)** | **P** | **Category** |
| 41 | CHROMATIN_ASSEMBLY_OR_DISASSEMBLY | 1.17 | 0.2073922 | cell structure |
| 42 | ONE_CARBON_COMPOUND_METABOLIC_PROCESS | 1.17 | 0.0 | metabolism |
| 43 | SECRETORY_PATHWAY | 1.16 | 0.09034908 | immune response / regulation |
| 44 | VIRAL_INFECTIOUS_CYCLE | 1.16 | 0.0 | immune response / regulation |
| 45 | NEURON_APOPTOSIS | 1.16 | 0.19507186 | Other |
| 46 | VIRAL_GENOME_REPLICATION | 1.16 | 0.0 | immune response / regulation |
| 47 | NEGATIVE_REGULATION_OF_CELL_DIFFERENTIATION | 1.16 | 0.0 | cell differentiation |
| 48 | SKELETAL_MUSCLE_DEVELOPMENT | 1.16 | 0.0 | cell differentiation |
| 49 | POSITIVE_REGULATION_OF_CYTOKINE_BIOSYNTHETIC_PROCESS | 1.15 | 0.29774126 | immune response / regulation |
| 50 | VIRAL_REPRODUCTION | 1.15 | 0.0 | immune response / regulation |
| 51 | NEGATIVE_REGULATION_OF_TRANSLATION | 1.15 | 0.0 | transcription/translation |
| 52 | TRANSCRIPTION_COACTIVATOR_ACTIVITY | 1.15 | 0.0927835 | transcription/translation |
| 53 | TRANSCRIPTION_ACTIVATOR_ACTIVITY | 1.15 | 0.0 | transcription/translation |
| 54 | CYTOKINE_METABOLIC_PROCESS | 1.15 | 0.0 | metabolism |
| 55 | CYTOKINE_BIOSYNTHETIC_PROCESS | 1.15 | 0.0 | metabolism |
| 56 | INTERLEUKIN_BINDING | 1.15 | 0.29774126 | immune response / regulation |
| 57 | STEROID_BIOSYNTHETIC_PROCESS | 1.15 | 0.21560575 | metabolism |
| 58 | CALCIUM_ION_BINDING | 1.14 | 0.30020705 | signal transduction |
| 59 | REGULATION_OF_MYELOID_CELL_DIFFERENTIATION | 1.14 | 0.09034908 | cell differentiation |
| 60 | REGULATION_OF_TRANSLATION | 1.14 | 0.19507186 | transcription/translation |
