# Supplementary material for: Cell Fate Decisions in Malignant Hematopoiesis: Leukemia Phenotype Is Determined by Distinct Functional Domains of the MN1 Oncogene
Source: PLoS One. 2014 Nov 17;9(11):e112671. doi: 10.1371/journal.pone.0112671 (PMC4234417; doi:10.1371/journal.pone.0112671)
Supplement: Table S8 — Gene ontology gene sets enriched in MN1Δ1 cells compared to MN1 cells. (DOC) [file pone.0112671.s017.doc]

**Supplementary Tables**

**Cell fate decisions in malignant hematopoiesis: Leukemia phenotype is determined by distinct functional domains of the MN1 oncogene**

Courteney K. Lai^1,2^, Yeonsook Moon^3^, Florian Kuchenbauer^4,5^, Daniel T. Starzcynowski^6^, Bob Argiropoulos^7^, Eric Yung^1^, Philip Beer^1^, Adrian Schwarzer^8^, Amit Sharma^8^, Gyeongsin Park^9^, Malina Leung^1^, Grace Lin^1^, Sarah Vollett^1^, Stephen Fung^1^, Connie J. Eaves^1,2^, Aly Karsan^10,11^, Andrew P. Weng^1,11^, R. Keith Humphries^1,2#^, Michael Heuser^12#^

**Table S8. Gene ontology gene sets enriched in MN1Δ**1 cells compared to MN1 cells.

| **Rank** | **Gene Sets** | **Normalised Enrichment Score (NES)** | **P** | **Category** |
| --- | --- | --- | --- | --- |
| 1 | SYNAPTOGENESIS | 1.53 | 0.0 | cell structure |
| 2 | METALLOENDOPEPTIDASE_ACTIVITY | 1.27 | 0.0 | signal transduction |
| 3 | GLUTATHIONE_TRANSFERASE_ACTIVITY | 1.24 | 0.0 | signal transduction |
| 4 | PROTEIN_SECRETION | 1.24 | 0.0 | cell structure |
| 5 | G_PROTEIN_COUPLED_RECEPTOR_BINDING | 1.23 | 0.0 | signal transduction |
| 6 | VIRAL_REPRODUCTION | 1.23 | 0.0 | immune response / regulation |
| 7 | SODIUM_ION_TRANSPORT | 1.23 | 0.19411765 | signal transduction |
| 8 | GTPASE_BINDING | 1.22 | 0.0 | signal transduction |
| 9 | PROTEIN_AMINO_ACID_DEPHOSPHORYLATION | 1.22 | 0.0 | signal transduction |
| 10 | CHEMOKINE_RECEPTOR_BINDING | 1.22 | 0.0 | immune response / regulation |
| 11 | SMALL_GTPASE_BINDING | 1.22 | 0.0 | signal transduction |
| 12 | DEPHOSPHORYLATION | 1.22 | 0.0 | signal transduction |
| **Rank** | **Gene Sets** | **Normalised Enrichment Score (NES)** | **P** | **Category** |
| 13 | VIRAL_REPRODUCTIVE_PROCESS | 1.22 | 0.0 | immune response / regulation |
| 14 | CHEMOKINE_ACTIVITY | 1.21 | 0.0 | immune response / regulation |
| 15 | CYTOKINE_ACTIVITY | 1.21 | 0.0 | immune response / regulation |
| 16 | SECRETION | 1.20 | 0.0 | cell structure |
| 17 | LYTIC_VACUOLE | 1.20 | 0.20512821 | cell structure |
| 18 | LYSOSOME | 1.20 | 0.20512821 | cell structure |
| 19 | IMMUNE_EFFECTOR_PROCESS | 1.20 | 0.18627451 | immune response / regulation |
| 20 | MICROSOME | 1.20 | 0.0 | cell structure |
| 21 | VOLTAGE_GATED_CHANNEL_ACTIVITY | 1.20 | 0.19488189 | signal transduction |
| 22 | TRANSCRIPTION_COREPRESSOR_ACTIVITY | 1.20 | 0.0 | transcription/translation |
| 23 | EXOCYTOSIS | 1.20 | 0.3019608 | cell structure |
| 24 | ACTIVATION_OF_IMMUNE_RESPONSE | 1.20 | 0.18627451 | immune response / regulation |
| 25 | RESPONSE_TO_VIRUS | 1.20 | 0.29637095 | immune response / regulation |
| 26 | VACUOLE | 1.20 | 0.20392157 | cell structure |
| 27 | VIRAL_GENOME_REPLICATION | 1.20 | 0.08431373 | immune response / regulation |
| 28 | VESICULAR_FRACTION | 1.19 | 0.0 | cell structure |
| 29 | VIRAL_INFECTIOUS_CYCLE | 1.19 | 0.0 | immune response / regulation |
| 30 | MESODERM_DEVELOPMENT | 1.19 | 0.0 | cell differentiation |
| 31 | SECRETION_BY_CELL | 1.19 | 0.0 | immune response / regulation |
| 32 | TRANSCRIPTION_REPRESSOR_ACTIVITY | 1.18 | 0.0 | transcription/translation |
| 33 | TRANSFERASE_ACTIVITY_TRANSFERRING_ALKYL_OR_ARYLOTHER_THAN_METHYLGROUPS | 1.18 | 0.20512821 | signal transduction |
| 34 | REGULATION_OF_MAP_KINASE_ACTIVITY | 1.18 | 0.08382066 | signal transduction |
| 35 | UNFOLDED_PROTEIN_BINDING | 1.18 | 0.19215687 | signal transduction |
| 36 | ACTIN_FILAMENT_ORGANIZATION | 1.18 | 0.18627451 | cell structure |
| 37 | OXIDOREDUCTASE_ACTIVITY_GO_0016705 | 1.18 | 0.29637095 | signal transduction |
| 38 | ACETYLGLUCOSAMINYLTRANSFERASE_ACTIVITY | 1.17 | 0.286 | signal transduction |
| 39 | RNA_CATABOLIC_PROCESS | 1.17 | 0.10784314 | metabolism |
| 40 | CELLULAR_RESPIRATION | 1.17 | 0.39803922 | metabolism |
| **Rank** | **Gene Sets** | **Normalised Enrichment Score (NES)** | **P** | **Category** |
| 41 | GLYCOPROTEIN_METABOLIC_PROCESS | 1.17 | 0.20967741 | metabolism |
| 42 | TRANSCRIPTION_FACTOR_BINDING | 1.16 | 0.0 | transcription/translation |
| 43 | CARBOXYLESTERASE_ACTIVITY | 1.16 | 0.0 | signal transduction |
| 44 | NEGATIVE_REGULATION_OF_TRANSCRIPTION_DNA_DEPENDENT | 1.16 | 0.0 | transcription/translation |
| 45 | NEGATIVE_REGULATION_OF_RNA_METABOLIC_PROCESS | 1.16 | 0.0 | metabolism |
| 46 | SPLICEOSOME | 1.16 | 0.3019608 | transcription/translation |
| 47 | AEROBIC_RESPIRATION | 1.16 | 0.30392158 | metabolism |
| 48 | MONOOXYGENASE_ACTIVITY | 1.16 | 0.2882353 | signal transduction |
| 49 | ONE_CARBON_COMPOUND_METABOLIC_PROCESS | 1.16 | 0.19411765 | metabolism |
| 50 | ATPASE_ACTIVITY_COUPLED_TO_TRANSMEMBRANE_MOVEMENT_OF_IONS_PHOSPHORYLATIVE_MECHANISM | 1.16 | 0.19411765 | signal transduction |
| 51 | NEGATIVE_REGULATION_OF_SIGNAL_TRANSDUCTION | 1.16 | 0.10784314 | signal transduction |
| 52 | PROTEIN_FOLDING | 1.16 | 0.20392157 | metabolism |
| 53 | AMINO_SUGAR_METABOLIC_PROCESS | 1.15 | 0.0 | metabolism |
| 54 | INFLAMMATORY_RESPONSE | 1.15 | 0.19723865 | immune response / regulation |
| 55 | SECRETORY_PATHWAY | 1.15 | 0.08431373 | immune response / regulation |
| 56 | CARBON_CARBON_LYASE_ACTIVITY | 1.15 | 0.08704454 | signal transduction |
| 57 | GTPASE_ACTIVITY | 1.15 | 0.08431373 | signal transduction |
| 58 | CHROMATIN_ASSEMBLY | 1.15 | 0.08431373 | cell structure |
| 59 | ACTIN_CYTOSKELETON_ORGANIZATION_AND_BIOGENESIS | 1.15 | 0.09467456 | cell structure |
| 60 | TRANSCRIPTION_COFACTOR_ACTIVITY | 1.15 | 0.19411765 | transcription/translation |
